# Telomere lengths in women treated for breast cancer show associations with chemotherapy, pain symptoms, and cognitive domain measures: a longitudinal study

**DOI:** 10.1186/s13058-020-01368-6

**Published:** 2020-12-04

**Authors:** Areej A. Alhareeri, Kellie J. Archer, Han Fu, Debra E. Lyon, R. K. Elswick, Debra L. Kelly, Angela R. Starkweather, Lynne W. Elmore, Yahya A. Bokhari, Colleen K. Jackson-Cook

**Affiliations:** 1 Department of Human & Molecular Genetics, Virginia Commonwealth University, 737 North 5th Street, Biotech 8, Suite 104, Richmond, VA 23129 USA; 2 King Saud bin Abdulaziz University for Health Sciences, Riyadh, Saudi Arabia; 3 King Abdullah International Medical Research Center, Riyadh, Saudi Arabia; 4 Division of Biostatistics, The Ohio State University, Columbus, OH USA; 5 College of Nursing, University of Florida, Gainesville, FL USA; 6 Family and Community Health Nursing, School of Nursing, Virginia Commonwealth University, Richmond, VA USA; 7 University of Connecticut School of Nursing, Storrs, CT USA; 8 American Cancer Society, Atlanta, GA USA; 9 Department of Pathology, Virginia Commonwealth University, Richmond, VA USA; 10 Member of the Massey Cancer Center, Virginia Commonwealth University, Richmond, VA USA

**Keywords:** Telomere, Breast cancer, Chemotherapy-related cognitive dysfunction, Psychoneurologic symptoms, Chromosome-specific telomere lengths

## Abstract

**Background:**

Survival rates for breast cancer (BC) have improved, but quality of life post-diagnosis/treatment can be adversely affected, with survivors reporting a constellation of psychoneurological symptoms (PNS) including stress, anxiety, depression, pain, fatigue, sleep disturbance, and cognitive dysfunction.

**Methods:**

To assess a potential relationship between telomere length (TL) and the development/persistence of PNS, we longitudinally studied 70 women (ages 23–71) with early stage BC (I-IIIA) at 5 time-points: prior to treatment (baseline), the mid-point of their chemotherapy cycle, 6 months, 1 year, and 2 years following the initiation of chemotherapy.

Measures quantified included assessments of each of the PNS noted above and TL [using both a multiplex qPCR assay and a chromosome-specific fluorescence in situ hybridization (FISH) assay].

**Results:**

Variables associated with qPCR mean TLs were age (*p* = 0.004) and race (T/S ratios higher in Blacks than Whites; *p* = 0.019). Significant differences (mostly decreases) in chromosome-specific TLs were identified for 32 of the 46 chromosomal arms at the mid-chemo time-point (*p* = 0.004 to 0.049). Unexpectedly, the sequential administration of doxorubicin [Adriamycin], cyclophosphamide [Cytoxan], and docetaxel [Taxotere] (TAC regimen) was consistently associated with higher TLs, when compared to TLs in women receiving a docetaxel [Taxotere], Carboplatin [Paraplatin], and trastuzumab [Herceptin] [TCH] chemotherapy regimen [association was shown with both the qPCR and FISH assays (*p* = 0.036)]. Of the PNS, pain was significantly negatively associated with TL (higher pain; shorter telomeres) for a subset of chromosomal arms (5q, 8p, 13p, 20p, 22p, Xp, Xq) (*p* = 0.014–0.047). Chromosomal TLs were also associated with 7 of the 8 cognitive domains evaluated, with the strongest relationship being noted for chromosome 17 and the visual memory domain (shorter telomeres; lower scores).

**Conclusions:**

We showed that race and age were significantly associated with telomere length in women treated for early stage BC and that acquired telomere alterations differed based on the woman’s treatment regimen. Our study also demonstrated that pain and cognitive domain measures were significantly related to telomere values in this study cohort. Expanding upon the knowledge gained from this longitudinal study could provide insight about the biological cascade of events that contribute to PNS related to BC and/or its treatment.

**Supplementary information:**

The online version contains supplementary material available at 10.1186/s13058-020-01368-6.

## Introduction

Breast cancer (BC) is the most common neoplasia observed in women, with approximately 1 in 8 women receiving a diagnosis of BC in their lifetime [1]. Due to improvements in screening tools, detection strategies, and treatment options, survival rates associated with BC have increased (90% of women with an early diagnosis survive at least five years) [1]. However, many of these survivors report the acquisition and persistence of side effects from their cancer treatment (or the cancer itself) that adversely affect their quality of life [2, 3]. These side effects include (but are not limited to) anxiety, stress, depression, pain, fatigue, sleep disturbance, and cognitive dysfunction, collectively categorized as “psychoneurological symptoms” (PNS) [4]. The acquisition and retention of PNS not only impacts treatment decisions/compliance in patients with BC, but can also challenge the cancer survivor’s ability to maintain employment, connect with loved ones, and carry out day-to-day tasks [5].

Although there is increased awareness of the importance for understanding the biological basis of cancer treatment-related PNS, the molecular mechanism(s) contributing to their development and persistence remain(s) enigmatic. Perturbations in inflammation processes have been conjectured to contribute to these PNS [6]. However, the fact that inflammatory molecules, such as cytokines, are not biologically retained for long periods of time, coupled with the observation that these PNS can persist in cancer survivors for years after therapy, led us to hypothesize that inflammation may be an early step in a cascade of biological events that also includes the acquisition of somatic cell genetic (e.g., telomere length changes; chromosomal instability; acquired point mutations) and/or epigenetic (e.g., methylation) changes. These genetic/epigenetic alterations could provide a means for the effects of the BC and/or its treatments to be maintained in an individual’s biological “memory” for months to years following treatment [7].

Of the above noted genetic alterations, telomeres are a particularly intriguing candidate for an association with PNS. Telomeres, which are nucleoprotein structures comprised of tandem repetitive arrays of a (TTAGGG)_n_ sequence and the shelterin complex, are located at the terminal ends of all vertebrate chromosomes, including those of humans [8]. Acquired telomere attrition has been speculated as a causal factor in the acquisition of many chronic diseases [9]. Furthermore, accelerated telomere shortening has been shown to play a role in the pathogenesis of multiple behavioral conditions, including (but not limited to) stress, depression, and cognitive decline, all of which are side effects that can be experienced by patients with BC [10].

Telomere length alterations in patients with BC have been evaluated as a diagnostic marker in breast tissues, but few investigators have evaluated the effect of chemotherapy on the telomere lengths of healthy, peripheral tissues in these patients [11]. Based on the scientific premise that telomere shortening is accelerated by inflammation and oxidative stress, and that chemotherapy affects both of these biological processes, the primary objective of this study was to longitudinally evaluate telomere length in women receiving treatment for BC over a 2-year period. The primary hypothesis for this study is that chemotherapy contributes to chromatin breakage/misrepair within the telomere, thereby leading to telomere alterations. In turn, these telomere alterations are hypothesized to mediate, or be associated with, a biological cascade of changes that lead to the acquisition/persistence of a subset of chemotherapy-related, acquired PNS.

## Materials and methods

### Ethics statement

This research involving human subjects was approved by the Virginia Commonwealth University Human Subjects’ Institutional Review Board (IRB # HM13194 CR4). Written documentation of informed consent was obtained from all study participants.

### Study participant ascertainment

For this study, 77 women with early stage (I to IIIA) BC were ascertained through 5 regional cancer centers in Central Virginia as part of the EPIGEN study, as previously described [12]. The study participant eligibility criteria were as follows: (1) 21 years of age or older, (2) a diagnosis of early stage BC with a scheduled visit to receive chemotherapy, and (3) a female gender (due to the low frequency of BC in males, only females were evaluated). Exclusion criteria were a history of (1) a previous cancer or chemotherapy, (2) a diagnosis of dementia, (3) active psychosis, or (4) an immune-related diagnosis (to avoid confounding due to inflammation). The five time-points in this longitudinal study were as follows: time-point 1 (“baseline” time-point, which was prior to the inception of chemotherapy), time-point 2 (“mid-chemo” time-point, which was prior to the fourth cycle of chemotherapy), time-point 3 (approximately 6 months following the inception of chemotherapy, at which time a subset of women received radiotherapy), and time-points 4 and 5, (approximately 1 year or 2 years following chemotherapy inception, respectively). Participants received either “dose dense” chemotherapy (every 2 weeks) or had an every 3-week chemotherapy administration (based on their regimen). All participants completed chemotherapy prior to time-point 3. After completion of chemotherapy, women with hormone sensitive tumors began hormonal agents.

During each time-point visit, blood specimens were collected from each study participant and transported to the laboratory for cell culture or DNA extraction. Telomere length assessments were measured using (1) a monochrome, multiplex real-time quantitative polymerase chain reaction (MMqPCR) method and (2) a chromosome arm-specific fluorescent in situ hybridization (FISH) technique. The lab personnel were blinded to the clinical history/chemotherapy status of the study participants at the time of sample processing and evaluation.

### Demographics, lifestyle, and clinical health information

For each study participant, demographic and lifestyle information was obtained (via self-reporting at time-point 1), along with clinical health information (extracted from the electronic health record following assessments by a research nurse), and tumor pathology information (included [but was not limited to] BC stage, BC grade, and tumor characteristics). Tumors were categorized (based on FISH and/or immunohistochemical testing results) as luminal A (ER+ and/or PR+; HER2−; no information was available on Ki-67), luminal B (ER+ and/or PR+; HER2+; no information was available on Ki-67), HER2 positive (ER−, PR−, and HER2+), and triple negative (ER−, PR−, and HER2−) [13].

### DNA isolation and monochrome multiplex qPCR (MMqPCR)

Genomic DNA was extracted from whole blood using a Puregene DNA Isolation Kit (Qiagen) according to the manufacturer’s protocol. After extraction, all specimens (100%) were quantified using a nanodrop, assessed for OD260/OD280 values (required a value of 1.8 or higher), and stored at − 80 °C in Qiagen DNA Hydration Solution (with storage times ranging from 1 month to 4.75 years).

The mean genomic telomere length of the study participants was calculated for the 5 time-points over the 2-year longitudinal study using a MMqPCR assay, as previously described [14]. Prior to setting up the MMqPCR reactions, aliquots from the DNA stocks were (1) evaluated to ensure the DNA was not degraded (using a Quibit assessment of the percentage of double-stranded DNA) and (2) diluted to approximately 20 ng in pure water. Each reaction well (of a 96-well plate) was set up by combining telomere primers

(telg [ACACTAAGGTTTGGGTTTGGGTTTGGGTTTGGGTTAGTGT] and telc [TGTTAGGTATCCCTATCCCTATCCCTATCCCTATCCCTAACA]), single copy housekeeping gene primers for human albumin

(albu [CGGCGGCGGGCGGCGCGGGCTGGGCGGaaatgctgcacagaatccttg] and albd [GCCCGGCCCGCCGCGCCCGTCCCGCCGgaaaagcatggtcgcctgtt]), aliquots of specimen DNA [20 ng (in 5 μL of molecular grade water)], and 15 μL of SYBR Green Mastermix (BioRad) [14]. The telomere (T) and single copy housekeeping gene (S) values were calculated using a 5-point standard curve, the latter of which was derived from serial dilutions (1.235 to 100 ng) of a “cocktail” DNA solution obtained from mixing equal quantities of DNA collected from blood specimens from 11 healthy women (using an identical protocol to that used for study participants). Aliquots from the same “cocktail” mix were used for all MMqPCR plates. Each 96-well plate was set up to include triplicate (3X) assessments of (1) the standard “cocktail” DNA, (2) study participant DNA, (3) negative controls (wells lacking DNA), and (4) DNA from three positive controls having “known” telomere lengths (MCF-7 cells [short telomeres]; a 35-year-old healthy female [JMS; moderate telomeres]; and a subclone of HeLa cells [long telomeres]). The T/S ratio values for these control specimens were assessed for each plate to ensure that each control showed small (MCF7), moderate (JMS), or large (HeLa) T/S values or no amplification product (blank wells) before including the proband values from that batch in the dataset for analysis. To reduce potential batch effects, all five time-points from each participant were evaluated from the same 96-well plate. As an additional layer of quality control, all reagents used for the MMqPCR studies were obtained from one single lot (no reagent lot differences).

The MMqPCR assay was run on a BioRad CFX96, with the data being quantified and T/S ratios calculated using BioRad CFX Manager software (version 3.0). The cycling parameters used were as follows: 95 °C for 5 min; 2 cycles of 94 °C for 15 s, 49 °C for 15 s; 49 cycles of 15 s at 94 °C, 15 s at 62 °C, 15 s at 83 °C, 15 s at 60 °C with signal acquisition, 15 s at 94 °C, 20 s at 85 °C with signal acquisition. The 60 °C reads provided the C_t_ values for the amplification of the telomere template (in early cycles when the albumin signal is still at baseline). The 85 °C reads provided the C_t_ values for the amplification of albumin template, at which time the telomere template is fully melted.

The raw telomere data for the triplicate measures for each study participant were reviewed. Prior to the statistical analyses, if any of the T/S ratio values showed greater than 10% variance from the other 2 values, this measure was excluded, with the mean T/S ratio being re-calculated using only two values [the outlier was excluded]). If more than 10% variance was observed between all 3 measures, the assay was repeated due to failure to meet quality control standards.

### Chromosome arm-specific telomere FISH

For the chromosome-specific telomere studies, leukocytes were isolated from blood specimens. Following stimulation using phytohemagglutinin, lymphocytes at the metaphase portion of the cell cycle were harvested at 72 h using standard procedures [15]. To assess telomere lengths, microscope slides containing metaphase chromosomes were prepared and hybridized with a pan-telomeric FITC-labeled peptide nucleic acid (PNA) probe [(CCCTAA)_3_], according to the manufacturer’s protocol (DakoCytomation, Denmark), with the DNA in these cells being visualized by counterstaining using a 5:1 DAPI II/propidium iodide (Abbott Molecular) solution.

**Fig. 1:**
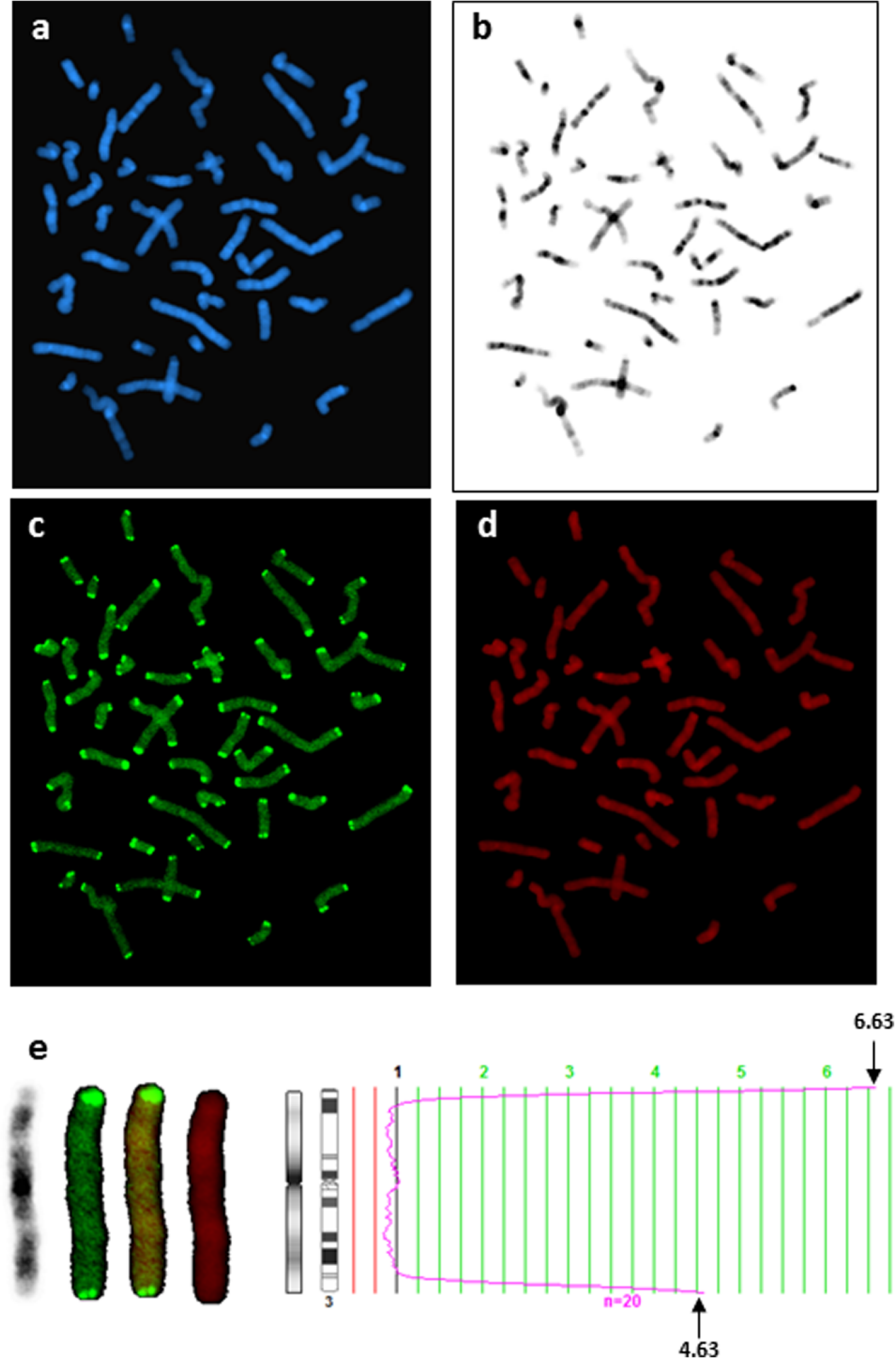
Fluorescence in situ hybridization (FISH) chromosome-specific telomere images. **a**–**d** The steps used for the chromosome-specific telomere assay. Individual chromosomes from each metaphase cell were evaluated based on information gained from their DAPI counterstaining appearance (**a**), which was used to generate a reverse DAPI banding image (**b**). The intensity/size of the telomere probe signal (**c**) was also evaluated, with a propidium iodide (PI) stain being used to delineate the chromosomal boundaries (**d**). **e** Based on the collective information gained from the reverse DAPI, FITC labeled, merged, and propidium iodide (PI) images (shown from left to right), ratio profiles were calculated for each chromosome. In image **e**, a representative chromosome 3 shows a short arm telomere (top of chromosome) that is larger (fluorescence intensity value of 6.63) than the long arm telomere (bottom of chromosome; value of 4.63)

Chromosome arm-specific telomere measures were completed for time-point 1 and time-point 2 for all study participants whose specimens yielded a sufficient number of metaphase chromosomes. The intensity values of the telomeric probe signals were calculated for each chromosome arm by averaging the values from a total of 10 metaphase spreads (20 homologs) per specimen using software on a Cytovision Leica Biosystems station (the CGH program), as previously described [15] (Fig. 1). Chromosomes that overlapped at the telomere region were omitted from the analysis to avoid any compromise in telomere probe signal quantification. Similarly, metaphase spreads that lacked sufficient banding for unequivocal chromosomal identification were excluded from analysis.

### Assessment of PNS

The PNS for the study participants were evaluated at each of the 5 time-points over a 2-year period, as described previously [16]. Briefly, cognitive function was evaluated using the CNS Vital Signs (CNSVS) computerized neurocognitive testing system [17]. The cognitive domains evaluated include (1) psychomotor speed, (2) reaction time, (3) complex attention, (4) cognitive flexibility, (5) executive functioning, (6) visual memory, (7) verbal memory, and (8) overall memory. The subscales of the CNSVS have been shown to have good test–retest reliability [17]. Stress was evaluated using the Perceived Stress Scale (PSS) [18]. Depressive symptoms and anxiety were evaluated using the Hospital Anxiety and Depression Scale (HADS) [19]. The remaining PNS assessed were pain, which was evaluated using the Brief Pain Inventory [20], fatigue, which was measured using the Brief Fatigue Inventory [21], and sleep disturbances, which were gauged using the General Sleep Disturbance Scale [22].

### Statistical analyses

#### Assessments of quality metrics for telomere assays

The reproducibility of the MMqPCR assay was assessed via an intraclass correlation coefficient analysis using a single-measurement, absolute-agreement, 2-way mixed-effects model [23, 24]. For comparisons between the two assays, it is important to recognize that there are differences in the manner in which telomeres are quantified between the MMqPCR and FISH methods. The MMqPCR assay provides a T/S ratio value that represents an “average” telomere length across all types of cells in a whole blood specimen relative to the value obtained for copies of albumin (a single copy gene). In contrast, the FISH method provides single cell information derived from lymphocytes for each individual telomere (chromosome arm specific) and has the potential to identify ends lacking a telomere (or having a very small telomere) [25]. Due to these methodological differences, one-to-one alignments of values derived from the two assays are not fully appropriate, nor are they expected to show full agreement. However, to compare the measures from the two different assays, the mean telomere value (averaged over all telomere ends and all cells) from the FISH method was compared to the T/S value using a Spearman’s rank correlation.

#### Assessments of telomere measures—MMqPCR assay telomere values

To determine associations between MMqPCR-based telomere length and breast cancer pathology/treatment, a mixed effects linear model was fit using an approach proposed by Hosmer and Lemeshow [26, 27]. In the first stage of the model building process, a base model was selected that represented the design of data collection and the timing of treatments (chemotherapy and radiation). Fixed effects included time-point, chemotherapy regimens, time-point by chemotherapy interaction (chemotherapy was administered only during time-point 2), radiation (yes/no), time-point by radiation interaction (radiation was only administered proximal to time-point 3), and a random effect for study participants. In the second stage, each variable was fit individually with the base model and if the *p* value was 0.25 or less, that variable was used in the third stage. Potential associated factors tested included demographic variables (age, race, and income), tumor characteristics (grade, stage, luminal A, luminal B, triple negative and HER2 positive status), and neoadjuvant status. In the third stage, all potential regressors (*p ≤* 0.25) were included into a multivariable mixed effects linear model. This initial model was further refined by sequentially removing variables from the model with the highest *p* values (backward stepwise) until all remaining variables had a *p* value of 0.05 or less. This model was considered the final model. Least squares means of significant associated variables were tested using Tukey’s test.

#### Assessments of telomere measures—chromosome-specific assay telomere values

For the chromosome-specific telomere assessments, a paired *t* test was used to compare mean telomere intensity values for each chromosomal arm [short (p) arm and long (q) arm] between baseline and mid-chemotherapy time-points. The percent change in telomere intensity values for each chromosomal arm between time-points was determined for each participant using the formula:

$$ \frac{\left(\mathrm{Mid}-\mathrm{chemo}\ \mathrm{telomere}\ \mathrm{intensity}\ \mathrm{value}-\mathrm{Baseline}\ \mathrm{telomere}\ \mathrm{intensity}\ \mathrm{value}\right)}{\mathrm{Baseline}\ \mathrm{telomere}\ \mathrm{intensity}\ \mathrm{value}}\times 100 $$

Negative values reflected a decrease in telomere length at the mid-chemotherapy compared to baseline time-points, a zero value reflected no change in telomere length, and a positive value reflected an increase in telomere length at the mid-chemotherapy compared to baseline time-points.

To identify significant variables associated with alterations in chromosome-specific telomere values, the previous base model was used, with the exception of excluding the radiation variable (since the chromosome-specific data did not include time-points following the administration of radiation). In addition, instead of using a backward stepwise approach, each of the above-mentioned potential variables associated with chromosome-specific telomere length was fit individually. A variable was identified as having a significant association when the analysis yielded a *p* value of 0.05 or less.

#### Assessment of relationship between telomere measures and cognitive function

Linear mixed effects models were used to examine the association between telomere length and cognitive functioning scores, while adjusting for potential confounders, which included individual characteristics (education, race, BMI), cancer-related traits (BC stage, estrogen receptor status, progesterone receptor status, Herceptin status), treatment-related factors (treatment regimens, surgery [adjuvant or neoadjuvant], radiation status), and depression as a concurrent symptom. The cognitive domains examined included psychomotor speed, reaction time, complex attention, cognitive flexibility, executive functioning, and visual, verbal, and overall memory.

Models assessing relationships of T/S values (MMqPCR assay) on the cognitive domain scores were established and fitted. Time was included using one year as the unit of measurement so that its magnitude would not overly influence inferences if higher orders of time were included in the models [0 (time-point 1), 4/52 (time-point 2), 0.5 (time-point 3), 1 (time-point 4), and 2 (time-point 5) years]. Due to the nonlinear trends in the cognitive scores observed from the data, the quadratic term of time was included such that both fixed effects and random effects included the intercept and time. Backward selection was performed to identify covariates for each cognitive domain. Backward elimination of random-effect terms was conducted using likelihood ratio tests with a significance threshold of 0.1, followed by elimination of fixed-effect terms by *F* tests, with a significance threshold of 0.05. The Satterthwaite method was used to compute the denominator degrees of freedom and *F* statistics [28].

The relationship of chromosome-specific telomere length on cognition was examined by replacing the T/S ratio (MMqPCR) telomere variable with the chromosome-specific telomere measurement variables, while maintaining all covariates that remained after the backward elimination procedure. Since the data on chromosome-specific telomere values were only available for the first two time-points, the squared time term was manually removed from the fixed effects and linear/squared time terms from the random effects, with the random intercept effect being retained in the new model. Mixed effects models and backward elimination procedures were implemented using *lmerTest* R package [29]. Two strategies were used to assess chromosome-specific telomere assay variables. The first was to introduce the telomere values of the two arms (p and q arm) of a chromosome together into the model and examine the combined effect of telomere values of the two arms by contrast tests. The second approach was to add telomere values one arm at a time and examine the significance using *t* tests. The latter approach was deemed most appropriate since the length of telomeres on each of the two arms of a chromosome are not always correlated [30]. For chromosomal arm-specific analyses, we calculated the false discovery rate (FDR) using the Benjamini-Hochberg method to adjust for the multiple hypothesis tests performed [31].

## Results

### Recruitment and demographics

In total, 77 women with early stage BC were recruited for this study. Five participants did not complete the study (4 withdrew due to a “lack of time/interest”; one developed osteomyelitis after time-point 2, requiring her exclusion per study criteria). Thus, telomere length assessments were initiated for a total of 72 study participants (93.5% retention rate).

Demographic data, breast tumor characterizations, and treatment information for these 72 women are shown in Table 1 and Additional File 1. Only two women (one in the Black cohort; one in the White cohort) self-reported having Latino/Hispanic ethnicity. Given this small number, no statistical analyses were completed for the Latino/Hispanic sub-group, with these women being included in the Black or White sub-groups, respectively. The age of the 72 women in the study ranged from 23 to 71 years, with a median age of 52 years. A significant difference in age was observed between the Black (mean = 46.6 years, standard error (s.e.) = 2.07 years) and White (mean = 53.5 years, s.e. = 1.37 years) participants (*p* = 0.007). Annual income also differed significantly between the two racial groups, with Whites having higher incomes than Blacks *(p* < 0.0001) (Table 1).

**Table 1:** Subset of demographics and chemotherapy regimens for study participants receiving treatment for breast cancer

Treatment/demographic factor	Black participants ***N*** = 22 (30.6%)				White participants ***N*** = 50 (69.4%)			
Therapy regimen	TAC^1^ *n* = 10 (45%)^2^	TC^1^ *n* = 6 (27%)	TCH^1^ *n* = 6 (27%)	Total *n* = 22 (100%)	TAC^1^ *n* = 29 (58%)	TC^1^ *n* = 15 (30%)	TCH^1^ *n* = 6(12%)	Total *n* = 50(100%)
**Age**	44.0 [3.4]^3^	47.0 [2.5]	50.5 [2.9]	46.6 [2.1]	52.2 [1.7]	57.1 [2.6]	51.0 [5.2]	53.5 [1.4]
**Income**								
Less than $30,000	5 (23%)	4 (18%)	3 (14%)	12 (55%)	6 (12%)	1 (2%)	0 (0%)	7 (14%)
$30,000–$59,999	3 (14%)	2 (9%)	3 (14%)	8 (37%)	5 (10%)	1 (2%)	0 (0%)	6 (12%)
$60,000–$89,999	1 (4%)	0 (0%)	0 (0%)	1 (4%)	8 (16%)	7 (14%)	3 (6%)	18 (36%)
$90,000+	1 (4%)	0 (0%)	0 (0%)	1 (4%)	10 (20%)	6 (12%)	3 (6%)	19 (36%)

^1^TAC = sequential administration of docetaxel (Taxotere), doxorubicin (Adriamycin), and cyclophosphamide (Cytoxan); TC = docetaxel (Taxotere) and cyclophosphamide (Cytoxan); TCH = docetaxel (Taxotere), Carboplatin (Paraplatin), and trastuzumab (Herceptin)

^2^Number in parentheses is the percentage of participants in this category (%)

^3^Mean and [standard error] for age is shown, respectively

### Tumor characteristics

No significant difference in the grade (*p* = 0.197) or stage (*p* = 0.055) of the BC tumors was noted in the Black compared to White study participants. While there was a trend toward a higher proportion of luminal A tumors and a lower proportion of HER2+ and triple negative tumors in the White participants (60% luminal A tumors; 10% luminal B tumors; 6% HER2+; 24% triple negative tumors) compared to Black participants (36% luminal A; 9% luminal B tumors; 18% HER2+; 36% triple negative tumors), these distributions were not significantly different (Supplementary Table 1, Additional File 1).

### Treatment

Three main types of chemotherapy regimens were administered to the study participants: (1) sequential administration of doxorubicin (Adriamycin), cyclophosphamide (Cytoxan), and docetaxel (Taxotere) [TAC], [54%]; (2) docetaxel (Taxotere) and cyclophosphamide (Cytoxan) [TC], [29%]; and (3) docetaxel (Taxotere), Carboplatin (Paraplatin), and trastuzumab (Herceptin) [TCH], [17%]. Two women received a cyclophosphamide, methotrexate and 5 fluorouracil treatment. Due to the small size of this latter group, these two participants were excluded from the study prior to the analysis of data. This exclusion resulted in a total sample size of 70 women for the data analyses. No significant differences in neoadjuvant/adjuvant status (*p* = 0.123), chemotherapy regimens (*p* = 0.291), or radiation treatment (*p* = 0.229) were observed between the racial groups (Table 1 and Supplementary Table 1, Additional File 1).

### Telomere measure quality metrics

The DNA extracted from each of the 350 specimens obtained from the 70 women evaluated (5 specimens per participant) met quality and quantity standards (100% pass rate). The intraclass correlation coefficient for the MMqPCR telomere assay was 0.933, with a 95% confidence interval range of 0.920 to 0.944. This value is categorized as showing excellent reliability, using the criteria of Koo and Li [23]. Comparisons between the triplicate measures of T and S values did not identify any “outlier” measures per study criteria. Thus, the mean T/S ratio was calculated using 3 replicate measures for all 350 specimens.

For the FISH chromosome-specific assay, baseline specimens were successfully completed for 73.6% of the study participants, with the majority of unsuccessful results being related to specimen collection challenges during the initiation of the study [insufficient specimens were collected for 19 participants]. Also, as noted above, 1 participant was excluded from the study due to her receipt of a cyclophosphamide, methotrexate, and 5 fluorouracil treatment (sample size too small for analysis)]. Only 4 specimens failed to yield results that were unrelated to specimen collections or study exclusion criteria (*n* = 2 at time-point 2; *n* = 1 at both time-points 1 and 2). After exclusions for incomplete data, paired chromosome-specific telomere length comparisons (T1 to T2) were completed for a subset of 50 women [*n* = 13 (26%) participants who self-identified as Black; *n* = 37 (74%) participants who self-identified as White].

The “mean” telomere value was calculated for the FISH assay (“pooled” data from all 46 individual telomeres across each of the single lymphocyte cells evaluated) for comparisons to the data derived from the genome-wide MMqPCR assay (which, as noted above, is completed on DNA extracted from whole blood and provides a mean value that is standardized in relation to a single copy locus evaluated [albumin]). The Spearman’s correlation calculated for “average” telomere lengths using these two different assays yielded a value of 0.275 (*p* = 0.058) at visit 1. Moreover, when estimating Spearman’s correlation for each chromosome and arm at visit 1, the value was significant for 13/46 (28.3%) tests at *p* ≤ 0.05 level, and significant for 26/46 (56.5%) tests at the *p* ≤ 0.10 level. Due to methodological differences, we did not expect to see a strong association between these values. This observation could reflect differences related to technical or stochastic factors, as well as biological variance (such as telomere free ends [detectable by FISH but not MMqPCR] or elongated telomeres arising from an alternative lengthening of telomeres (ALT) process [more readily detected by the FISH method than the MMqPCR method]).

### Associations with telomere length values estimated using MMqPCR

**Fig. 2:**
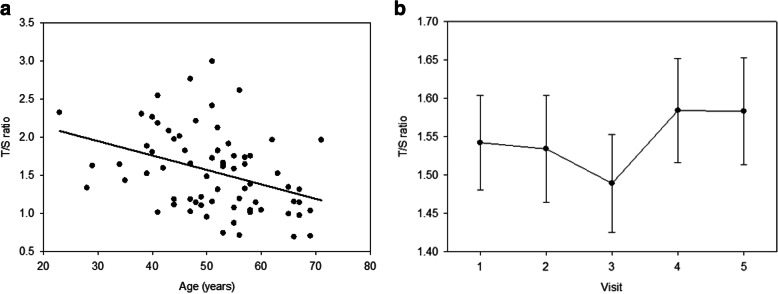
MMqPCR telomere data. **a** Average genomic telomere values at baseline were negatively correlated with age (*r* = − 0.37, *p* = 0.001 shown graphically by the trendline). Each circle (•) represents a study subject. Age is shown on the *X*-axis, with the baseline T/S ratio value being shown on the *Y*-axis. **b** Average genomic telomere lengths for the 72 women evaluated are shown at baseline (time-point 1) (mean = 1.542, s.e. = 0.062), mid-chemo (time-point 2) (mean = 1.534, s.e. = 0.070), time-point 3 (mean = 1.489, s.e. = 0.064), time-point 4 (mean = 1.584, s.e. = 0.068), and time-point 5 (mean = 1.583, s.e. = 0.070). The vertical bars represent standard error values associated with each mean

Using a Pearson’s correlation, a significant negative correlation was detected between the participants’ age and their T/S value in their baseline specimens (shorter telomeres with older age; *r* = − 0.37, *p* = 0.001) (Fig. 2a). Potential relationships between T/S values and treatment, demographic variables, or PNS were evaluated by fitting the data into a mixed effects linear regression model (Additional File 2). The final optimized model identified age (*p* = 0.004), race (*p* = 0.019), and the type of chemotherapy treatment (*p* = 0.044) as having a significant association with mean telomere (T/S) values. The estimated parameter for age was − 0.0134; thus, for every 1 year increase in age, there is a corresponding 0.0134 decrease in T/S ratio. For the racial groups, T/S values were higher in the Black (mean = 1.661; s.e. = 0.087) compared to White participants (mean = 1.420; s.e. = 0.062). Study visit time was not significantly associated with T/S ratios (*p* = 0.650) (Fig. 2b), but the type of chemotherapy used for treatment was significantly associated with telomere length (*p* = 0.045), with the women receiving TAC treatment having significantly higher T/S values (mean = 1.682; s.e. = 0.074) than the women receiving TCH (mean = 1.366; s.e. = 0.103) regimens (*p =* 0.036) using Tukey’s test (Additional File 2). However, the telomere values for women receiving TC treatment were not significantly different from those receiving TCH (*p* = 0.273) or TAC (*p* = 0.536) regimens. No other significant associations were observed between T/S ratios and the other variables evaluated in this model across the five time-points, including a lack of association with the PNS of anxiety (*p* = 0.461), depression *p* = 0.633), pain (0.946), fatigue (0.759), sleep disturbance (0.825), or stress (0.760) (Additional File 2).

### Associations with telomere length values estimated using chromosome-specific FISH

**Fig. 3:**
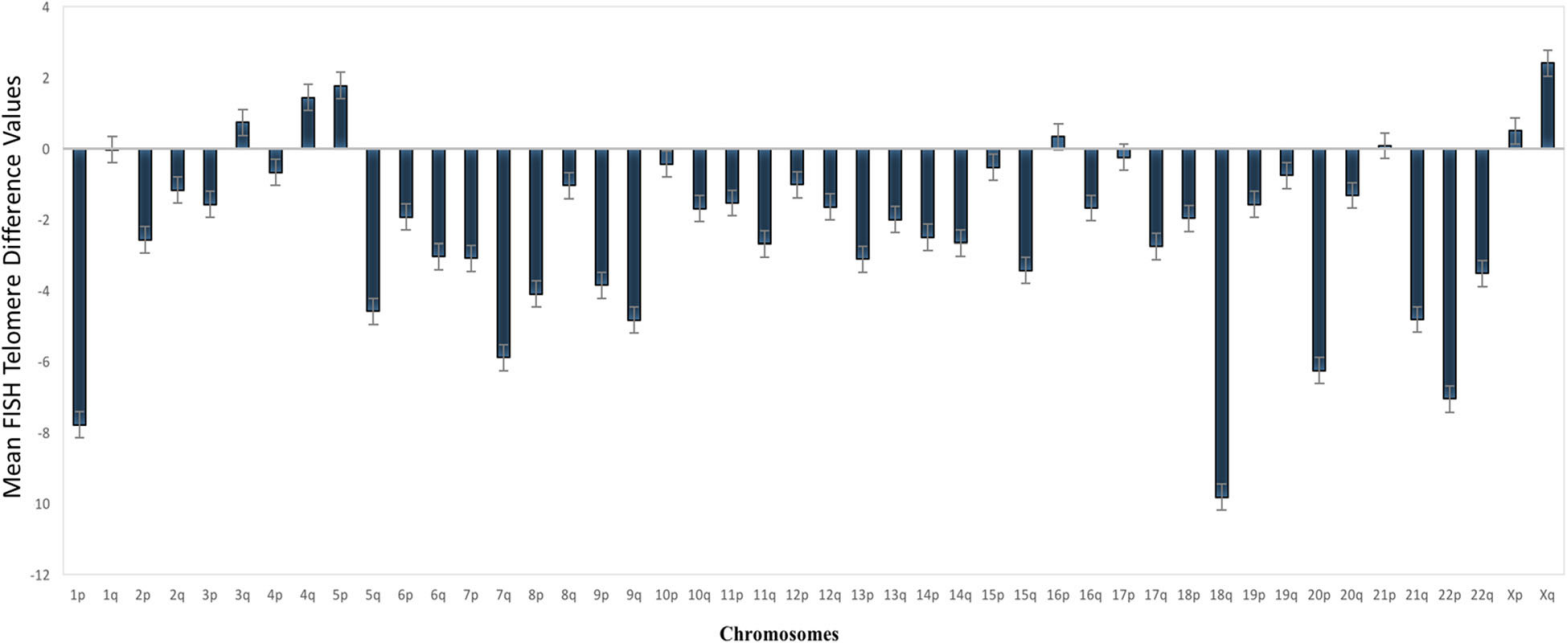
Mean changes in chromosome-specific telomere values at baseline (T1) compared to mid-chemotherapy cycle (T2) Time-points. A negative value (shortening) was observed at the mid-chemo time-point for 38 of the 46 chromosomal arms. The *X*-axis shows values for each chromosomal arm. The *Y*-axis shows mean differences in telomere values. The bars on the histograms indicate standard errors. Abbreviations: p = short arm; q = long arm.

The FISH chromosome-specific statistical comparisons were completed based on the percent change in telomere values for each chromosomal arm (short arm and long arm) for the 50 study participants yielding results (graphically represented in Fig. 3 and Additional File 3; demographic/pathology data for this subset is shown in Additional File 4). Based on the chromosome-specific FISH data, the majority of telomeres (84.8%) showed attrition (negative values), but 15.2% of telomeres showed increases in values (Fig. 3 Additional File 5).

At the mid-chemotherapy time-point, a mixed effects model, adjusted for age, showed significant shortening for 8 telomeres [1p (*p* = 0.022), 5q (*p* = 0.041), 7q (*p* = 0.025), 9q (*p* = 0.045), 18q (*p* = 0.002), 20p (*p* = 0.020), 21q (*p* = 0.040), and 22p (*p* = 0.025)], along with marginal significance values for decreases in length for 12 additional telomeres [6p, 6q, 7p, 8p, 9p, 10q, 13p, 13q, 14q, 17q, 18p, and 22q] (Additional Files 5 and 6).

To further assess the association between the types of chemotherapy treatments and telomere lengths, a fixed effects model, adjusted for age, was also fit to the chromosome-specific data. The type of chemotherapy treatment was associated with telomere lengths for 32 of the 46 chromosomal arms (69.6%; Table 2), with participants receiving TAC chemotherapy treatment showing larger mean telomere values compared to study participants receiving TC or TCH treatment.

**Table 2:** Mixed effects linear regression model fitting assessment of association of chemotherapy type with chromosome-specific telomere length

Chromosome arm	Least square mean			***p*** value	
	TAC^**1**^	TC	TCH		FDR
**1p**	4.346 (0.251)*	**2.944 (0.451)**	**2.813 (0.501)**	**0.004**	**0.048**
**1q**	**5.642 (0.314)**	**4.016 (0.564)**	**4.433 (0.626)**	**0.028**	**0.056**
**2p**	**5.602 (0.315)**	**3.929 (0.565)**	**4.174 (0.628)**	**0.017**	**0.048**
2q	4.247 (0.263)	3.125 (0.473)	3.486 (0.526)	0.094	0.101
3p	6.252 (0.319)	4.671 (0.574)	5.508 (0.637)	0.059	0.075
**3q**	**5.488 (0.317)**	**3.853 (0.570)**	**4.051 (0.634)**	**0.020**	**0.048**
4p	4.475 (0.280)	3.671 (0.502)	3.436 (0.558)	0.157	0.160
4q	5.876 (0.326)	4.425 (0.586)	4.689 (0.651)	0.059	0.075
**5p**	**5.451 (0.295)**	**3.696 (0.531)**	**4.376 (0.589)**	**0.014**	**0.048**
5q	4.590 (0.309)	3.682 (0.556)	3.714 (0.618)	0.064	0.080
**6p**	**5.698 (0.298)**	**4.028 (0.536)**	**4.417 (0.596)**	**0.015**	**0.048**
**6q**	**5.383 (0.312)**	**3.835 (0.561)**	**4.279 (0.623)**	**0.039**	**0.064**
**7p**	**4.598 (0.269)**	**3.465 (0.484)**	**3.383 (0.538)**	**0.043**	**0.066**
**7q**	**5.335 (0.305)**	**3.673 (0.549)**	**4.163 (0.609)**	**0.022**	**0.050**
**8p**	**5.453 (0.288)**	**3.821 (0.518)**	**4.301 (0.575)**	**0.016**	**0.048**
8q	4.685 (0.312)	3.252 (0.561)	3.593 (0.623)	0.056	0.075
**9p**	**5.664 (0.314)**	**4.201 (0.564)**	**4.304 (0.627)**	**0.034**	**0.058**
**9q**	**4.037 (0.259)**	**2.581 (0.465)**	**2.821 (0.517)**	**0.012**	**0.048**
**10p**	**5.729 (0.313)**	**4.114 (0.562)**	**4.107 (0.624)**	**0.013**	**0.048**
10q	4.821 (0.299)	3.726 (0.537)	3.531 (0.597)	0.070	0.083
11p	4.791 (0.292)	3.639 (0.524)	3.758 (0.582)	0.090	0.099
**11q**	**5.413 (0.327)**	**4.152 (0.587)**	**3.837 (0.653)**	**0.045**	**0.067**
**12p**	**4.890 (0.287)**	**3.289 (0.515)**	**3.918 (0.572)**	**0.023**	**0.050**
12q	4.838 (0.289)	3.674 (0.520)	3.791 (0.578)	0.083	0.093
**13p**	**5.093 (0.289)**	**3.422 (0.519)**	**4.096 (0.576)**	**0.018**	**0.048**
**13q**	**5.599 (0.323)**	**4.146 (0.580)**	**4.111 (0.645)**	**0.033**	**0.058**
**14p**	**5.221 (0.314)**	**3.349 (0.565)**	**3.864 (0.628)**	**0.010**	**0.048**
**14q**	**5.000 (0.312)**	**3.487 (0.561)**	**3.856 (0.623)**	**0.041**	**0.065**
**15p**	**4.885 (0.285)**	**3.131 (0.512)**	**3.896 (0.569)**	**0.012**	**0.048**
**15q**	**5.535 (0.318)**	**3.823 (0.571)**	**4.112 (0.634)**	**0.017**	**0.048**
**16p**	**4.064 (0.237)**	**2.993 (0.426)**	**3.137 (0.473)**	**0.049**	**0.070**
**16q**	**4.614 (0.281)**	**3.126 (0.504)**	**3.398 (0.560)**	**0.020**	**0.048**
17p	4.282 (0.250)	3.083 (0.449)	4.464 (0.499)	0.051	0.071
17q	4.265 (0.276)	3.126 (0.496)	3.507 (0.551)	0.110	0.115
18p	5.224 (0.322)	4.218 (0.578)	4.404 (0.643)	0.238	0.238
**18q**	**5.556 (0.292)**	**4.058 (0.526)**	**4.310 (0.584)**	**0.024**	**0.050**
**19p**	**4.281 (0.248)**	**2.280 (0.445)**	**3.185 (0.495)**	**0.010**	**0.048**
**19q**	**4.852 (0.261)**	**3.232 (0.469)**	**3.786 (0.522)**	**0.009**	**0.048**
20p	5.054 (0.300)	3.684 (0.539)	4.122 (0.599)	0.067	0.081
**20q**	**4.430 (0.262)**	**2.917 (0.470)**	**3.414 (0.522)**	**0.015**	**0.048**
**21p**	**5.356 (0.278)**	**3.474 (0.500)**	**4.242 (0.555)**	**0.005**	**0.048**
**21q**	**4.700 (0.257)**	**3.121 (0.462)**	**3.267 (0.513)**	**0.004**	**0.048**
**22p**	**5.068 (0.282)**	**3.438 (0.506)**	**3.995 (0.562)**	**0.016**	**0.048**
**22q**	**4.269 (0.264)**	**3.086 (0.475)**	**3.028 (0.527)**	**0.031**	**0.058**
Xp	5.518 (0.322)	4.320 (0.579)	4.190 (0.643)	0.077	0.089
**Xq**	**5.237 (0.316)**	**3.834 (0.567)**	**3.756 (0.630)**	**0.032**	**0.058**

*The standard error is shown in parenthesis; bold rows indicate values showing statistical significance using an FDR threshold

^1^TAC = sequential administration of docetaxel (Taxotere), doxorubicin (Adriamycin), and cyclophosphamide (Cytoxan); TC = docetaxel (Taxotere) and cyclophosphamide (Cytoxan); TCH = docetaxel (Taxotere), Carboplatin (Paraplatin), and trastuzumab (Herceptin)

### Relationships between telomere values and psychoneurological symptoms

Using the mixed effects model that was adjusted for age, pain was found to be significantly negatively associated with telomere values (higher pain; shorter telomeres) for 5q (*p* = 0.040), 8p (*p* = 0.047), 13p (*p* = 0.019), 20p (*p* = 0.036), 22p (*p* = 0.035), Xp (*p* = 0.014), and Xq (*p* = 0.039) (Table 3). However, significant associations were not detected between telomere values and anxiety, depression, fatigue, sleep disturbance, or stress (*p* > 0.05).

**Table 3:** Association of pain and chromosome-specific telomere values (higher pain levels associated with smaller telomere values)

	Parameter estimate	Standard error	***p*** value	FDR
Chromosome				
1p	− 0.165	0.099	0.103	0.180
1q	− 0.196	0.124	0.121	0.180
2p	− 0.127	0.126	0.319	0.326
2q	− 0.146	0.105	0.170	0.201
3p	− 0.185	0.127	0.151	0.193
3q	− 0.182	0.126	0.156	0.194
4p	− 0.131	0.122	0.248	0.265
4q	− 0.185	0.130	0.160	0.194
5p	− 0.188	0.117	0.115	0.180
**5q**	**− 0.120**	**0.120**	**0.040**	0.180
6p	− 0.131	0.120	0.279	0.292
6q	− 0.222	0.122	0.076	0.180
7p	− 0.197	0.105	0.069	0.180
7q	− 0.119	0.123	0.335	0.335
**8p**	**− 0.229**	**0.112**	**0.047**	0.180
8q	− 0.195	0.123	0.122	0.180
9p	− 0.242	0.122	0.054	0.180
9q	− 0.164	0.102	0.116	0.180
10p	− 0.199	0.124	0.114	0.180
10q	− 0.202	0.118	0.093	0.180
11p	− 0.177	0.116	0.133	0.180
11q	− 0.203	0.129	0.123	0.180
12p	− 0.175	0.113	0.130	0.180
12q	− 0.215	0.113	0.064	0.180
**13p**	**− 0.267**	**0.110**	**0.019**	0.180
13q	− 0.197	0.128	0.131	0.180
14p	− 0.158	0.126	0.215	0.241
14q	− 0.189	0.124	0.133	0.180
15p	− 0.186	0.112	0.106	0.180
15q	− 0.216	0.125	0.090	0.180
16p	− 0.168	0.093	0.077	0.180
16q	− 0.204	0.110	0.070	0.180
17p	− 0.164	0.099	0.103	0.180
17q	− 0.212	0.108	0.055	0.180
18p	−0.252	0.125	0.051	0.180
18q	−0.181	0.116	0.124	0.180
19p	− 0.145	0.098	0.148	0.193
19q	− 0.187	0.102	0.075	0.180
**20p**	**− 0.251**	**0.116**	**0.036**	0.180
20q	− 0.126	0.105	0.235	0.257
21p	− 0.185	0.110	0.099	0.180
21q	− 0.139	0.102	0.180	0.207
**22p**	**− 0.237**	**0.109**	**0.035**	0.180
22q	− 0.189	0.103	0.075	0.180
**Xp**	**− 0.311**	**0.122**	**0.014**	0.180
**Xq**	**− 0.260**	**0.122**	**0.039**	0.180

Bold rows indicate statistical significance (*p* ≤ 0.05)

**Fig. 4:**
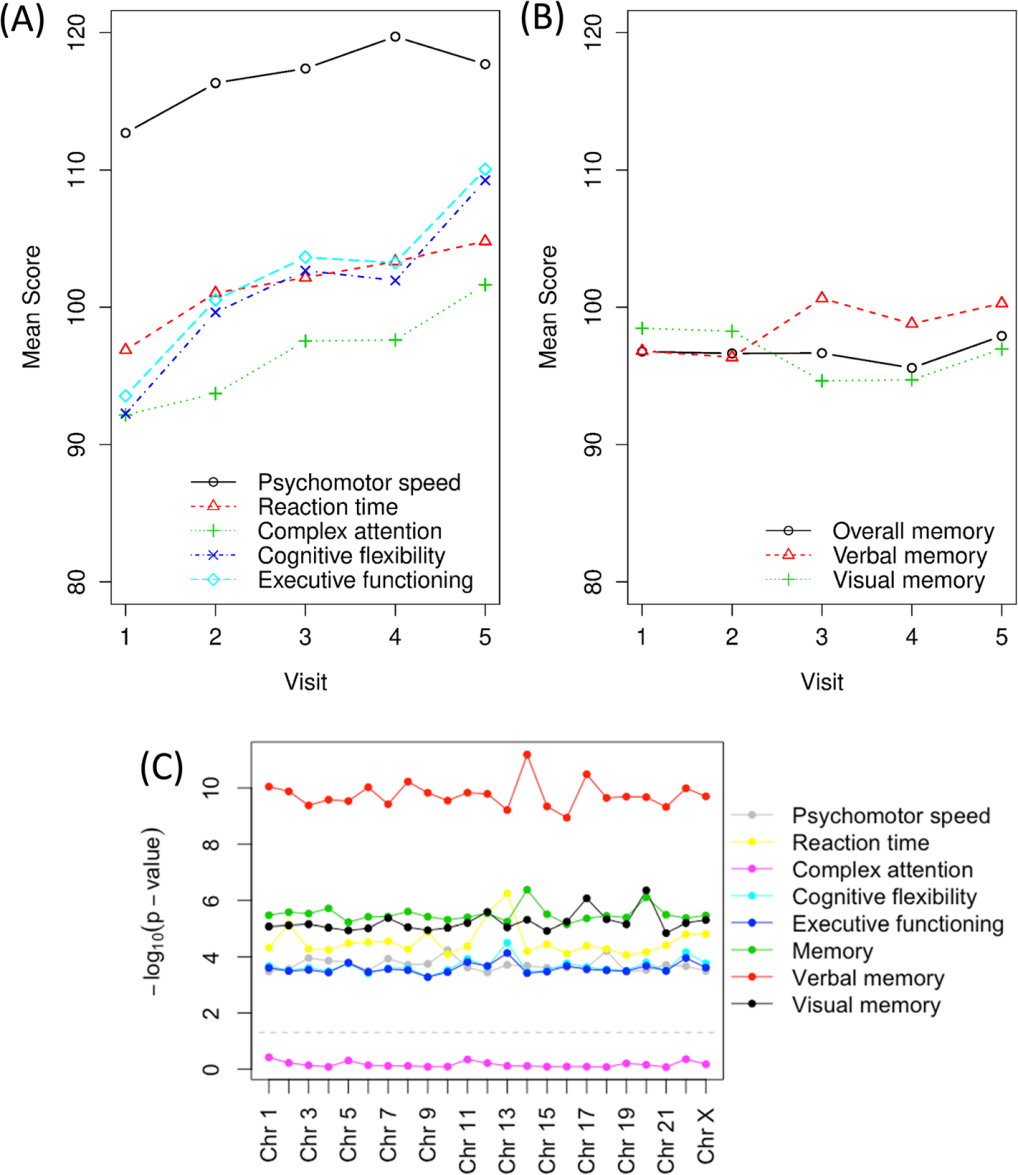
Assessments of telomere lengths and cognitive domains. **a** The trajectory of average standard scores (*Y*-axis) over time-point (*X*-axis) for each of the eight cognitive domains evaluated at each of the 5 time-points in this longitudinal study included: **a** speed, reaction time, complex attention, cognitive flexibility, executive function, and **b** memory domains. **c** The negative log_10_ of *p* values from contrast tests jointly evaluating the short arms and long arms for each chromosome. Each line represents one cognitive domain. The gray dashed line represents the corresponding value when the *p* value is 0.05

**Fig. 5:**
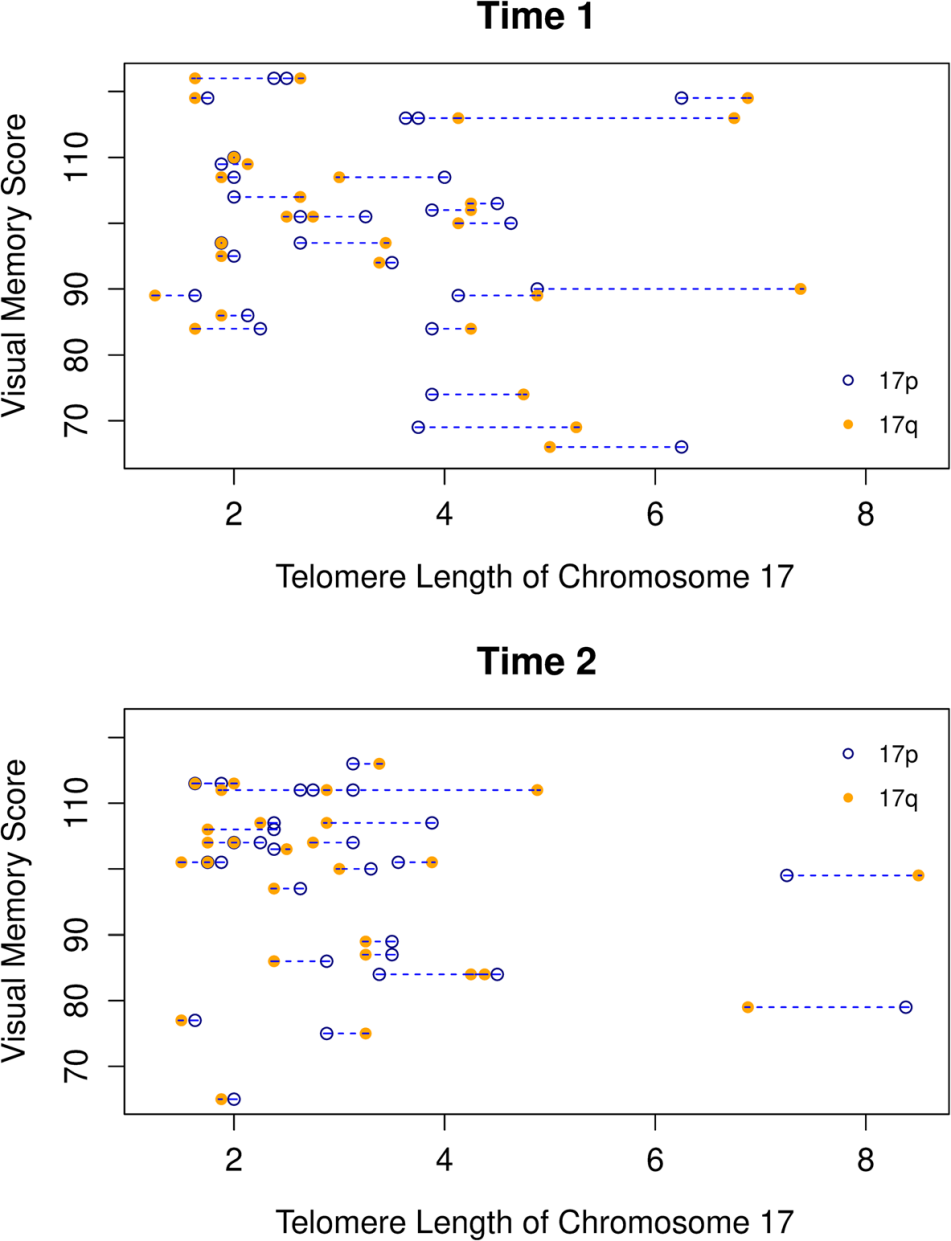
Associations between chromosome-specific telomere length and cognitive measures. Scatter plots of visual memory scores (*Y*-axis) against telomere lengths for chromosome 17 (*X*-axis) are shown, stratified by time-point. The chromosome 17 telomere values for the short arm (open blue circle) and long arm (solid orange circle) of each individual are connected by broken lines, with the telomere values being shown on the *X*-axis and the visual memory scores being shown on the *Y*-axis. Comparing the plots from the baseline data (time-point 1) (**a**) to those of the mid-chemo data (time-point 2) (**b**), one can see the generalized pattern of telomere shortening (shift of values to left) with decreases in visual memory scores (downward shift of values with tighter clustering)

To evaluate potential relationships between telomere values and cognition, plots of mean scores by time-point for each cognitive domain were visually examined to determine whether higher order terms for time should be incorporated in the longitudinal models (Fig. 4). The three memory domains showed modest changes along the time axis, while the other five domains displayed non-linear increasing trends over time. After backwards elimination of random and fixed effects, the covariates included in the models for each cognitive domain were identified. No significant associations were observed between the T/S values and the cognitive domains. However, an assessment of the chromosome-specific telomere values (in which the *p* values were obtained from contrast tests jointly evaluating the short arms and long arms for each chromosome) showed significant associations for seven of the eight cognitive domains (using a 0.05 significance level) (Table 4, Additional File 7). This finding is also visually demonstrated in Fig. 5, which shows the relationship between the visual memory domain and chromosome 17 (the chromosome that yielded the lowest *p* value among all of the chromosomes evaluated).

**Table 4:** Results of backward elimination and significance of FISH telomere values for each cognitive domain

Domain	Remaining fixed effects after backwards elimination in model	Remaining random effects^**1**^	Coefficient for MMqPCR telomere values (*p* value)	Significantly associated chromosomes using chromosome-specific values (*p* value < 0.5)
Psychomotor speed	Time, stage, progesterone positive, surgery, age, race	1 + time	1.93 (0.40)	All
Reaction time	Time, time, estrogen positive, HER positive, neoadjuvant, chemo final	1 + time	1.22 (0.40)	All
Complex attention	Time, estrogen positive, HER positive, chemo final, education	1	0.19 (0.94)	None
Cognitive flexibility	Time, HER positive, chemo final, age, race	1	1.14 (0.54)	All
Executive functioning	Time, HER positive, chemo final, age, race	1	1.21 (0.51)	All
Memory	Surgery, depression	1 + time	− 2.45 (0.18)	All
Verbal memory	Race, BMI	1	1.90 (0.39)	All
Visual memory	Surgery, education, depression	1	− 1.22 (0.53)	All

^1^In the third column, “1” represents random intercept and “time” represents random slop

Potential relationship between chromosome-specific telomere values and cognitive domains were also assessed using a model wherein each individual telomere was evaluated separately. This analytic approach showed significant relationships between visual memory domain scores and individual telomeres localized to (a) the long arm of chromosomes 1, 2, 3, 4, 6, 7, 8, 10, 11, 12, 13, 15, 16, 18, 20, 22, and X and (b) the short arm of chromosomes 3, 7, 8, 10, 11, 13, 14, 15, 16, 17, 18, and 19 (Additional File 7]). However, no significant associations were detected for other cognitive domains.

## Discussion

In this longitudinal study, we used two complementary assays to assess potential changes in telomere values in women following their receipt of chemotherapy for BC. The use of two assays provided an opportunity to identify both average (genome-wide) and single (potentially sentinel) telomere alterations that might be present. Consistent with the results of other investigators [32], age was associated with telomere length, with baseline telomere values showing a significant inverse correlation with age (older women had shorter telomeres). This observation is well-aligned with the canonical relationship observed between telomere attrition and the aging process (due to the end replication problem) [33].

In addition to age, race was also observed to be associated with mean telomere length in our study cohort, with Blacks having larger T/S ratio values (longer telomeres) than Whites. This observation has also been reported by other investigators studying the relationship of race on telomere length in women newly diagnosed with BC [34], as well as members of families with high/low risk for cardiovascular disease [35], and healthy individuals [36]. The mechanisms underlying potential racial differences in telomere length are not well understood, but have been conjectured to include possible differences in population genetic variance, cellular processes, and/or repair of oxidative damage levels [37, 38]. Although the mixed effects model used in this study was adjusted for age, due to the inter-relatedness of human epidemiology variables, one cannot fully rule out the possibility that the age distribution in our study cohort may have contributed to the observed association of race with telomere values, since the Black participants were significantly younger than the White participants.

To determine if chemotherapy was associated with changes in telomere length, we compared telomere values within individuals (before, during or after chemotherapy). Significant associations between chemotherapy regimen and telomere values were consistently observed with both the MMqPCR-based data and the chromosome-specific data, with both assays showing statistically significant changes in telomere values (increases) for the women who received a TAC regimen. In addition to this regimen-related finding, assessments of the chromosome-specific data (but not the MMqPCR data) showed significant telomere attrition (smaller values) for a subset of 8 different chromosomal arms; specifically, 1p, 5q, 7q, 9q, 18q, 20p, 21q, and 22p. Possible explanations for the observed discrepancy in detecting chromosomal shortening related to chemotherapy between the two telomere assays include (but are not limited to) (1) a lack of the MMqPCR assay to detect individual telomere heterogeneity (since the T/S ratio is an average value, the presence of relatively long telomeres could mask the presence of a sub-set of short telomeres present on specific chromosomal arms) and (2) telomeres that are extremely short/missing may be undetectable using the MMqPCR assay.

Few investigators have evaluated chromosome-specific telomere lengths as they relate to cancer and/or chemotherapy, with the few reported studies focusing on the relationship between chromosome-specific length and the risk of acquiring BC or esophageal cancer (Additional File 8). In their review of the effects of cancer treatment on telomere lengths, Gallicchio et al. [11] noted conflicting results between studies, with the observed variations reflecting differences in study designs/methodologies, as well as treatment regimens. The results of our investigation underscore the value of assessing telomere length using a longitudinal study design and using a telomere assay that provides estimates of individual telomeres, rather than a genomic “average” length (Additional File 8).

The mechanism(s) for how chemotherapy might alter telomere length are not fully known (Additional File 9) [39]. It is feasible that these drugs have a direct effect that alters (or leads to breakage of) the DNA in these regions, given that (1) telomeres have been shown to be particularly susceptible to oxidative damage and (2) repair of the damage incurred for this region is relatively inefficient [39–42]. For example, Adriamycin (also known as doxorubicin), which is in the TAC regimen, is a member of the anthracycline class of chemotherapeutic agents, the latter of which are thought to shorten telomeres through direct DNA damage by free radical formation [43, 44]. Another proposed mechanism of action for anthracyclines is the inhibition of DNA repair enzymes, particularly topoisomerase II, which may contribute to telomere shortening through further accumulation of DNA damage [43–45]. Alternatively, chemotherapeutic drugs that are frequently used in treating BC could have an indirect impact on telomeres by affecting members of the shelterin complex. Interestingly, doxorubicin has been found to reduce the expression of shelterin complex proteins POT1 and TPP1; in turn, a reduction in the expression of these proteins could alter the telomere cap structure and compromise its stability, thereby leading to telomere alterations [41, 42]. While a subset of women receiving TAC had shortened telomeres, we also observed the unexpected result that, overall, patients on the TAC regimen had longer mean telomere lengths when compared to patients receiving TC or TCH regimens. Based on our previously published observation of an increased rate of chromosomal instability in these same women who received the TAC regimen [12] (who showed significantly higher micronuclei frequencies in women receiving the TAC regimen), a plausible conjecture for this observation is that an alternative lengthening of telomeres (ALT) pathway was activated as a telomere maintenance mechanism following either (1) extensive DNA damage/breakage caused by exposure to TAC [46] or (2) uncapping of telomeres that were no longer able to bind sufficient shelterin proteins [47]. While little is known about ALT in healthy peripheral tissues following chemotherapy, it is becoming evident that cancer cells within a single tumor may adapt to rely on hTERT and/or ALT to restore/maintain critically shortened telomeres [48]. However, the mechanism(s) underlying this process is/are not fully understood, including whether ALT may preferentially act on specific telomeres.

Another fundamental question addressed in this study was the potential association between telomere length and PNS in patients treated for early stage BC. No statistically significant associations between PNS and overall mean telomere length were observed using the MMqPCR method, when collectively evaluated across all study participants at all 5 time-points. However, our chromosome-specific telomere length data identified a significant association between perceived pain and telomere length for a subset of chromosomes (5q, 8p, 13p, 20p, 22p, Xp, and Xq) at time-point 2 (mid-point of chemotherapy cycle). Significant associations between chromosome-specific telomere values and most of the cognitive domains evaluated (except complex attention) were also detected for time-point 2 (mid-point of chemotherapy cycle).

The biological nature of the associations between pain and/or cognitive domains and telomere length is unclear. It is possible that this relationship is merely correlative, with the telomere length serving as a “canary in a coal mine” type observation that enables one to document that a biologically relevant change has occurred. Alternatively, rather than serving as a “passive” indicator, it is feasible that telomere alterations actively contribute to aspects of cognitive dysfunction and/or pain via downstream cellular consequences (Additional File 10) [7, 49]. Briefly, chemotherapy for breast cancer (or the cancer itself) can lead to telomere breakage (with telomere repair or replication potentially being compromised by nucleotide depletion related to DNA damage repair throughout the genome) [50]. This can lead to alterations in telomere length or function, which, in turn, can lead to chromosomal instability. At least a portion of the chromosomal instability events (micronuclei and/or DNA telomeric fragments) that might arise following chemotherapy would be detected by the cGAS-STING pathway, thereby triggering the innate immune surveillance pathway [51–54]. This process targets the cells for senescence, the latter of which also contributes to inflammation (via senescence-associated secretory phenotype), thereby perpetuating the telomere alteration-chromosomal instability-inflammation cycle. The resulting cellular senescence could lead to compromises in tissue homeostasis. Moreover, telomere alterations (and inflammation) could contribute to epigenetic alterations, the latter of which could lead to changes in gene expression. Collectively, or singly, these biological changes related to telomere alterations (senescence; epigenetic changes; altered gene expression) could contribute to gene/cell functional changes that could contribute to the acquisition and/or persistence of PNS [7, 55].

A strength of our study is its longitudinal design, which allowed for the assessment of telomere length prior to chemotherapy, during active treatment and into survivorship. Moreover, most studies on telomere dynamics focus on measuring average genomic telomere length using a qPCR-based method. Our use of a chromosome-specific assay enabled us to assess individual (potentially sentinel) telomeres. However, since high quality metaphases for the chromosome-specific assay could not be obtained from all study subjects at all time-points, the sample size was decreased for this “high resolution” and labor intensive assay. Similarly, while more than 350 specimens were evaluated in this longitudinal study, the data were derived from only 70 individuals. Thus, further studies evaluating a larger number of individuals seem warranted to assess the potential for broader applicability of these findings.

## Conclusions

In summary, we showed that race and age were significantly associated with telomere shortening in women treated for early stage BC. Using our chromosome-specific assay, we also showed that most telomeres in lymphocytes obtained from women treated for BC showed attrition following the initiation of chemotherapy treatment, but that the change in telomere length (from pretreatment values) differed based on the type of chemotherapy they received. In particular, the TAC regimen was consistently associated with increases in telomere values based on data gained from both a MMqPCR and a chromosome-specific assay. Lastly, our study showed that pain and cognitive domain measures were significantly related to chromosome-specific (possibly sentinel) telomere values in this study cohort. If confirmed, the results of this study suggest that further research focusing on telomeres in healthy peripheral tissues could provide insight about the biological cascade of events that contribute to PNS related to BC and/or its treatment.

## Supplementary Information

**Additional file 1.** Breast Tumor Characteristics for Complete Group of Study Participants. List of pathology and treatment findings for the complete cohort of study participants.

**Additional file 2. **a. Mixed Effects Linear Model Fitting Assessment of Associations of Variables with MMqPCR Telomere Length and b. Mixed Effects Linear Model Fitting Assessment for Variables NOT Showing Significant MMqPCR Telomere Length Associations. Tables show least square means and *p* values for variables evaluated in the mixed effects linear model.

**Additional file 3.** Differences in Baseline (T1) Compared to Mid-Chemotherapy (T2) Time-points for FISH-based Chromosome-Specific Telomere Values. Heatmap showing differences in individual telomere values for each participant.

**Additional file 4.** a. Breast Tumor Characteristics for Subset of 50 Study Participants Evaluated using the Chromosome-Specific FISH Method and b. Demographics and Chemotherapy Regimens for Subset of 50 Study Participants Evaluated using the Chromosome-Specific FISH Method. List pathology and treatment findings for the subset of 50 participants evaluated with the chromosome-specific analysis at time-points 1 and 2.

**Additional file 5. **Comparisons of Chromosome-Specific Telomere Values Between Baseline and Mid-Chemo Specimens. List of the means, standard deviations, mean of difference between baseline and time-point 2 and the *p* values for each individual telomere (short arm 1, long arm 1, etc) calculated using the FISH chromosome-specific assay.

**Additional file 6. **Mixed Effects Linear Model Fitting Assessment of Mid-Chemo Timepoint as a Variable Associated with Chromosome-Specific Telomere Length. List of least square values, standard errors, and *p* values for results of mixed effects linear model fitting of chromosome-specific data.

**Additional file 7.** False Discovery Rates of Contrast Tests Jointly Evaluating the Short Arm and Long Arm Telomere Values for Each Chromosome With Cognitive Domains. Statistical values are shown for comparisons completed between cognitive domain scores and chromosome-specific telomere values.

**Additional file 8.** Chromosome-Specific Telomere Length and Cancer Risk. Literature review of previous reports of chromosome-specific telomere lengths related to cancer.

**Additional file 9.** Studies on the Effect of Doxorubicin (Adriamycin) on Telomere Biology. Literature review of previous reports assessing the biological response/mechanism of Doxorubincin (also called Adriamycin) on telomeres.

**Additional file 10.** Hypothesized Biological Cascade Contributing to Psychoneurological Symptoms in Women Receiving Chemotherapy for Breast Cancer. Schematic representation of inter-relationships between telomeres and inflammation, which lead to psychoneurological symptoms via alterations in epigenetic patterns/gene expression, and/or senescence.

## Data Availability

The datasets analyzed during the current study are available from the corresponding author on reasonable request.
