# Supplementary material for: Telomere lengths in women treated for breast cancer show associations with chemotherapy, pain symptoms, and cognitive domain measures: a longitudinal study
Source: Breast Cancer Res. 2020 Dec 4;22:137. doi: 10.1186/s13058-020-01368-6 (PMC7716505; doi:10.1186/s13058-020-01368-6)
Supplement: Supplementary file 1 — Additional file 1. Breast Tumor Characteristics for Complete Group of Study Participants. List of pathology and treatment findings for the complete cohort of study participants. [file 13058_2020_1368_MOESM1_ESM.docx]

**Additional File 1. Breast Tumor Characteristics for Complete Group of Study Participants**

| **Tumor or treatment attribute** | **Blacks**  **n=22**  **(31.4%)** | **Whites**  **n=50**  **(71.4%)** |
| --- | --- | --- |
|  |  |  |
| **Luminal A** |  |  |
| Yes | 8 (36%) | 30 (60%) |
| No | 14 (64%) | 20 (40%) |
| **Luminal B** |  |  |
| Yes | 2 (9%) | 5 (10%) |
| No | 20 (91%) | 45 (90%) |
| **Triple Negative** |  |  |
| Yes | 8 (36%) | 12 (24%) |
| No | 14 (64%) | 38 (76%) |
| **HER2 Positive** |  |  |
| Yes | 4 (18%) | 3 (6%) |
| No | 14 (64%) | 38 (76%) |
| **Grade** |  |  |
| 1 | 1 (4%) | 4 (8%) |
| 2 | 12 (55%) | 16 (32%) |
| 3 | 9 (40%) | 30 (60%) |
| **Stage** |  |  |
| I | 5 (22%) | 15 (30%) |
| IIA | 11 (50%) | 19 (38%) |
| IIB | 6 (28%) | 8 (16%) |
| IIIA | 0 (0%) | 8 (16%) |
| **Neoadjuvant** |  |  |
| Yes | 4 (18%) | 3(6%) |
| No | 18 (82%) | 47 (94%) |
| **Herceptin** |  |  |
| Yes | 6 (27%) | 8 (16%) |
| No | 5 (23%) | 42 (84%) |
| **Radiation** |  |  |
| Yes | 19 (87%) | 37 (74%) |
| No | 3 (13%) | 13 (26%) |

*****Number of participants (percentage [rounded to whole numbers] of participants in this category
