# Supplementary material for: Telomere lengths in women treated for breast cancer show associations with chemotherapy, pain symptoms, and cognitive domain measures: a longitudinal study
Source: Breast Cancer Res. 2020 Dec 4;22:137. doi: 10.1186/s13058-020-01368-6 (PMC7716505; doi:10.1186/s13058-020-01368-6)
Supplement: Supplementary file 2 — Additional file 2. a. Mixed Effects Linear Model Fitting Assessment of Associations of Variables with MMqPCR Telomere Length and b. Mixed Effects Linear Model Fitting Assessment for Variables NOT Showing Significant MMqPCR Telomere Length Associations. Tables show least square means and p values for variables evaluated in the mixed effects linear model. [file 13058_2020_1368_MOESM2_ESM.docx]

**Additional File 2 a. Mixed Effects Linear Model Fitting Assessment of Associations of Variables with MMqPCR Telomere Length**

|  | **Final Model** | |  | |  | |
| --- | --- | --- | --- | --- | --- | --- |
| **Variable** | **Least Squares Mean** | **Std. Err.** | | **p-value** | |  |
| **Time-point** |  |  | | 0.650 | |  |
| 1 – Baseline | 1.502 | 0.080 | |  | |  |
| 2 – Mid chemo | 1.520 | 0.080 | |  | |  |
| 3 – 6 months post chemo inception | 1.495 | 0.080 | |  | |  |
| 4 – 1 year post chemo inception | 1.588 | 0.080 | |  | |  |
| 5 – 2 years post chemo inception | 1.601 | 0.081 | |  | |  |
| **Chemotherapy^1^**  TAC  TC  TCH | 1.682  1.575  1.366 | 0.074  0.088  0.103 | | 0.045* | |  |
| **Chemotherapy by time-point** |  |  | | 0.264 | |  |
| TAC – Time-point 1 | 1.615 | 0.102 | |  | |  |
| TAC – Time-point 2 | 1.693 | 0.102 | |  | |  |
| TAC ­– Time-point 3 | 1.679 | 0.102 | |  | |  |
| TAC – Time-point 4 | 1.808 | 0.103 | |  | |  |
| TAC – Time-point 5 | 1.614 | 0.104 | |  | |  |
| TC – Time-point 1 | 1.506 | 0.126 | |  | |  |
| TC – Time-point 2 | 1.435 | 0.126 | |  | |  |
| TC – Time-point 3 | 1.630 | 0.126 | |  | |  |
| TC – Time-point 4 | 1.598 | 0.126 | |  | |  |
| TC – Time-point 5 | 1.708 | 0.127 | |  | |  |
| TCH – Time-point 1 | 1.384 | 0.150 | |  | |  |
| TCH – Time-point 2 | 1.432 | 0.150 | |  | |  |
| TCH – Time-point 3 | 1.176 | 0.150 | |  | |  |
| TCH – Time-point 4 | 1.358 | 0.150 | |  | |  |
| TCH – Time-point 5 | 1.482 | 0.155 | |  | |  |
| **Radiation** |  |  | | 0.880 | |  |
| Yes | 1.533 | 0.056 | |  | |  |
| No | 1.549 | 0.094 | |  | |  |
| **Radiation by Time-point** |  |  | | 0.265 | |  |
| Yes – Time-point 1 | 1.579 | 0.080 | |  | |  |
| Yes – Time-point 2 | 1.530 | 0.080 | |  | |  |
| Yes – Time-point 3 | 1.410 | 0.080 | |  | |  |
| Yes – Time-point 4 | 1.512 | 0.080 | |  | |  |
| Yes – Time-point 5 | 1.634 | 0.083 | |  | |  |
| No – Time-point 1 | 1.424 | 0.134 | |  | |  |
| No – Time-point 2 | 1.509 | 0.134 | |  | |  |
| No – Time-point 3 | 1.580 | 0.134 | |  | |  |
| No – Time-point 4 | 1.664 | 0.134 | |  | |  |
| No – Time-point 5 | 1.568 | 0.134 | |  | |  |
| **Age^2^** | 51.3 | 1.231 | | 0.004* | |  |
| **Race**  Blacks  Caucasians | 1.661  1.420 | 0.087  0.062 | | 0.019* | |  |

* Variables significantly predictive of telomere length

^1^TAC = Sequential administration of docetaxel (Taxotere), doxorubicin (Adriamycin), & cyclophosphamide (Cytoxan); TC = Docetaxel (Taxotere) and cyclophosphamide (Cytoxan); TCH = Docetaxel (Taxotere), Carboplatin (Paraplatin), and trastuzumab (Herceptin)

^2^The estimated parameter for Age was –0.0134, thus, for every 1 year increase in age, there is a corresponding 0.0134 decrease in T/S ratio.

**b. Mixed Effects Linear Model Fitting Assessment for Variables NOT Showing Significant MMqPCR Telomere Length Associations**

| **Variable** | **Base Model p value^1^** |
| --- | --- |
| Tumor Stage | 0.148 |
| Tumor Grade | 0.954 |
| Herceptin Yes/No | 0.857 |
| Neoadjuvant | 0.506 |
| Luminal A | 0.891 |
| Luminal B | 0.529 |
| Triple Negative | 0.997 |
| HER2 Positivity | 0.350 |
| Anxiety | 0.461 |
| Depression | 0.633 |
| Sleep disturbance | 0.825 |
| Fatigue | 0.759 |
| Stress | 0.760 |

^1^ The base model, which was determined by the study design, was: telomere length = Visit +

Chemotherapy (3 types) + Radiation therapy +Visit by chemotherapy3 + Visit by Radiation

therapy with the study subject being the random effect

The final model reflects variables that remained significantly associated with telomere length

after stepwise removals. The results of the final model are presented in Supplementary Table S2a.
