# Supplementary material for: Telomere lengths in women treated for breast cancer show associations with chemotherapy, pain symptoms, and cognitive domain measures: a longitudinal study
Source: Breast Cancer Res. 2020 Dec 4;22:137. doi: 10.1186/s13058-020-01368-6 (PMC7716505; doi:10.1186/s13058-020-01368-6)
Supplement: Supplementary file 3 — Additional file 3. Differences in Baseline (T1) Compared to Mid-Chemotherapy (T2) Time-points for FISH-based Chromosome-Specific Telomere Values. Heatmap showing differences in individual telomere values for each participant. [file 13058_2020_1368_MOESM3_ESM.docx]

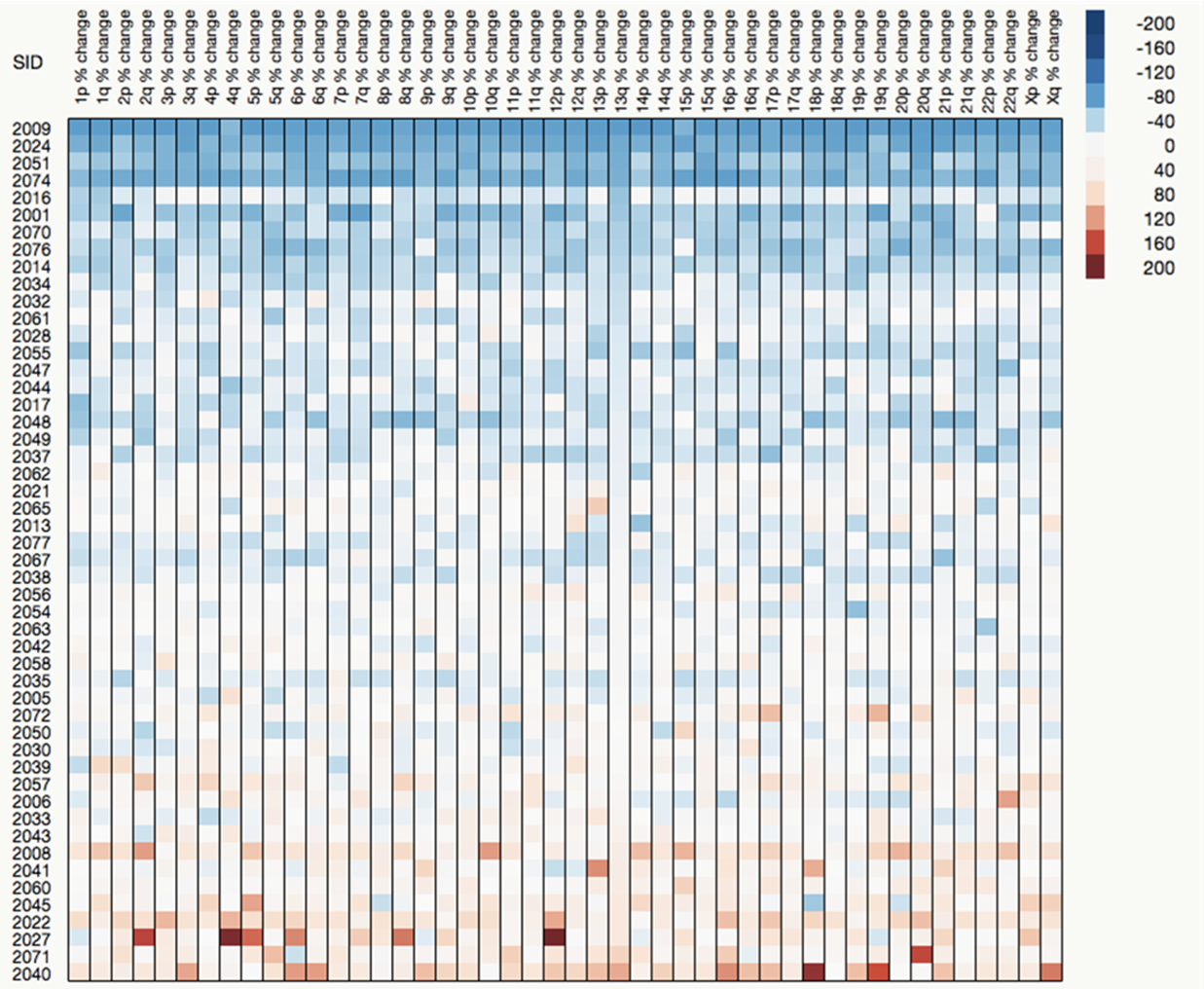


**Additional File 3. Differences in Baseline (T1) Compared to Mid-Chemotherapy (T2) Time-points for FISH-based Chromosome-Specific Telomere Values.** This heat map shows the percent changes in telomere intensity values from pre-treatment (time-point 1) compared to the mid-point of the chemotherapy treatment (time-point 2). Each column represents a chromosomal arm and each row represents a study participant [SID=Study Identifier]. The legend shows the percent change, with decreases in telomere length being represented by negative values (shown in blue hues), whereas increases in telomere length are represented by positive values (shown in red hues).
