# Supplementary material for: Telomere lengths in women treated for breast cancer show associations with chemotherapy, pain symptoms, and cognitive domain measures: a longitudinal study
Source: Breast Cancer Res. 2020 Dec 4;22:137. doi: 10.1186/s13058-020-01368-6 (PMC7716505; doi:10.1186/s13058-020-01368-6)
Supplement: Supplementary file 4 — Additional file 4. a. Breast Tumor Characteristics for Subset of 50 Study Participants Evaluated using the Chromosome-Specific FISH Method and b. Demographics and Chemotherapy Regimens for Subset of 50 Study Participants Evaluated using the Chromosome-Specific FISH Method. List pathology and treatment findings for the subset of 50 participants evaluated with the chromosome-specific analysis at time-points 1 and 2. [file 13058_2020_1368_MOESM4_ESM.docx]

**Additional File 4 a. Breast Tumor Characteristics for Subset of 50 Study Participants Evaluated using the Chromosome-Specific FISH Method.**

| **Tumor or treatment attribute** | **Blacks**  **n=13**  **(26.0%)** | **Whites**  **n=37**  **(74.0%)** |
| --- | --- | --- |
|  |  |  |
| **Luminal A** |  |  |
| Yes | 4 (31%) | 23 (62%) |
| No | 9 (69%) | 14 (38%) |
| **Luminal B** |  |  |
| Yes | 1 (8%) | 4 (11%) |
| No | 12 (92%) | 33 (89%) |
| **Triple Negative** |  |  |
| Yes | 6 (46%) | 8 (22%) |
| No | 7 (54%) | 29 (78%) |
| **HER2 Positive** |  |  |
| Yes | 2 (15%) | 2 (5%) |
| No | 11 (85%) | 35 (95%) |
| **Grade** |  |  |
| 1 | 0 (0%) | 3 8%) |
| 2 | 5 (38%) | 13 (35%) |
| 3 | 8 (62%) | 21 (57%) |
| **Stage** |  |  |
| I | 4 (31%) | 10 (27%) |
| IIA | 7 (54%) | 11 (30%) |
| IIB | 2 (15%) | 8 (22%) |
| IIIA | 0 (0%) | 8 (22%) |
| **Neoadjuvant** |  |  |
| Yes | 2 (18%) | 2 (5%) |
| No | 11 (82%) | 35 (95%) |
| **Herceptin** |  |  |
| Yes | 3 (23%) | 6 (16%) |
| No | 10 (77%) | 31 (84%) |
| **Radiation** |  |  |
| Yes | 11 (85%) | 27 (73%) |
| No | 2 (15%) | 10 (27%) |

**b. Demographics and Chemotherapy Regimens for Subset of 50 Study Participants Evaluated using the Chromosome-Specific FISH Method.**

| **Treatment/**  **Demographic Factor** | **Black Participants**  **n=13 (26.0%)** | | | | **White Participants**  **n=37 (74.0%)** | | | |
| --- | --- | --- | --- | --- | --- | --- | --- | --- |
| **Therapy Regimen** | TAC^1^  n=6  (46%)^2^ | TC^1^  n=4  (31%) | TCH^1^  n=3  (23%) | Total  n=13  (100%) | TAC^1^  n=26  (70%) | TC^1^  n=6  (16%) | TCH^1^  n=5  (14%) | Total  n=37  (100%) |
| **Age** | 42.5  [3.0]^3^ | 47.5  [3.4] | 55.3  [3.7] | 47.0  [2.3] | 52.3  [1.9] | 58.3  [3.3] | 50.2  [6.4] | 53.0  [1.7] |
| **Income**  Less than $30,000 | 1 (8%) | 3 (23%) | 2 (15%) | 6 (46%) | 5 (14%) | 1 (3%) | 0 (0%) | 6(16%) |
| $30,000 - $59,999 | 3 (23%) | 1 (8%) | 1 (8%) | 5 (38%) | 5 (14%) | 1 (3%) | 0 (0%) | 6 (16%) |
| $60,000 - $89,999 | 1 (8%) | 0 (0%) | 0 (0%) | 1 (8%) | 6 (16%) | 3 (8%) | 3 (8%) | 12 (32%) |
| $90,000+ | 1 (8%) | 0 (0%) | 0 (0%) | 1 (8%) | 10 (27%) | 1 (3%) | 2 (5%) | 13 (35%) |

^1^TAC = Sequential administration of docetaxel (Taxotere), doxorubicin (Adriamycin), & cyclophosphamide (Cytoxan); TC = Docetaxel (Taxotere) and cyclophosphamide (Cytoxan); TCH = Docetaxel (Taxotere), Carboplatin (Paraplatin), and trastuzumab (Herceptin)

^2^Number in parentheses is the percentage of participants in this category (%)

^3^Mean and [standard error] for age is shown, respectively
