# Supplementary material for: Telomere lengths in women treated for breast cancer show associations with chemotherapy, pain symptoms, and cognitive domain measures: a longitudinal study
Source: Breast Cancer Res. 2020 Dec 4;22:137. doi: 10.1186/s13058-020-01368-6 (PMC7716505; doi:10.1186/s13058-020-01368-6)
Supplement: Supplementary file 5 — Additional file 5. Comparisons of Chromosome-Specific Telomere Values Between Baseline and Mid-Chemo Specimens. List of the means, standard deviations, mean of difference between baseline and time-point 2 and the p values for each individual telomere (short arm 1, long arm 1, etc) calculated using the FISH chromosome-specific assay. [file 13058_2020_1368_MOESM5_ESM.docx]

**Additional File 5. Comparisons of Chromosome-Specific Telomere Values Between Baseline and Mid-Chemo Specimens**
