# Supplementary material for: Telomere lengths in women treated for breast cancer show associations with chemotherapy, pain symptoms, and cognitive domain measures: a longitudinal study
Source: Breast Cancer Res. 2020 Dec 4;22:137. doi: 10.1186/s13058-020-01368-6 (PMC7716505; doi:10.1186/s13058-020-01368-6)
Supplement: Supplementary file 6 — Additional file 6. Mixed Effects Linear Model Fitting Assessment of Mid-Chemo Timepoint as a Variable Associated with Chromosome-Specific Telomere Length. List of least square values, standard errors, and p values for results of mixed effects linear model fitting of chromosome-specific data. [file 13058_2020_1368_MOESM6_ESM.docx]

**Additional File 6. Mixed Effects Linear Model Fitting Assessment of Mid-Chemo Timepoint as a Variable Associated with Chromosome-Specific Telomere Length**

| **Chromosome Arm** | **Least Square Mean at Baseline** | | **Least Square Mean at Mid-Chemo** | | **Std. Error** | **p-value** |
| --- | --- | --- | --- | --- | --- | --- |
| 1p | 3.632 | | | 3.103 | 0.264 | **0.022** |
| 5q | | 4.428 | | 3.803 | 0.330 | **0.041** |
| 7q | | 4.757 | | 4.024 | 0.331 | **0.025** |
| 9q | | 3.376 | | 2.917 | 0.217 | **0.045** |
| 18q | | 5.078 | | 4.204 | 0.311 | **0.002** |
| 20p | | 4.635 | | 3.938 | 0.320 | **0.020** |
| 21q | | 3.954 | | 3.438 | 0.274 | **0.040** |
| 22p | | 4.499 | | 3.835 | 0.305 | **0.025** |
|  | |  | |  |  |  |
