# Supplementary material for: Telomere lengths in women treated for breast cancer show associations with chemotherapy, pain symptoms, and cognitive domain measures: a longitudinal study
Source: Breast Cancer Res. 2020 Dec 4;22:137. doi: 10.1186/s13058-020-01368-6 (PMC7716505; doi:10.1186/s13058-020-01368-6)
Supplement: Supplementary file 7 — Additional file 7. False Discovery Rates of Contrast Tests Jointly Evaluating the Short Arm and Long Arm Telomere Values for Each Chromosome With Cognitive Domains. Statistical values are shown for comparisons completed between cognitive domain scores and chromosome-specific telomere values. [file 13058_2020_1368_MOESM7_ESM.docx]

**Additional File 7. False Discovery Rates of Contrast Tests Jointly Evaluating the Short Arm and Long Arm Telomere Values for Each Chromosome With Cognitive Domains.**

|  | **Psychomotor Speed** | **Reaction Time** | **Complex Attention** | **Cognitive Flexibility** | **Executive Functioning** | **Memory** | **Verbal Memory** | **Visual Memory** |
| --- | --- | --- | --- | --- | --- | --- | --- | --- |
| **Chr 1** | 4.00E-04 | 4.00E-04 | 0.432 | 0.00033 | 0.00036 | 2.00E-05 | <0.00001 | 2.00E-05 |
| **Chr 2** | 0.00038 | 0.00038 | 0.662 | 4.00E-04 | 4.00E-04 | 2.00E-05 | <0.00001 | 2.00E-05 |
| **Chr 3** | 0.00021 | 0.00021 | 0.775 | 0.00036 | 4.00E-04 | 2.00E-05 | <0.00001 | 2.00E-05 |
| **Chr 4** | 0.00025 | 0.00025 | 0.830 | 4.00E-04 | 0.00042 | 1.00E-05 | <0.00001 | 3.00E-05 |
| **Chr 5** | 0.00028 | 0.00028 | 0.549 | 0.00029 | 0.00028 | 2.00E-05 | <0.00001 | 3.00E-05 |
| **Chr 6** | 4.00E-04 | 4.00E-04 | 0.775 | 0.00045 | 0.00042 | 2.00E-05 | <0.00001 | 3.00E-05 |
| **Chr 7** | 0.00021 | 0.00021 | 0.800 | 0.00036 | 0.00038 | 2.00E-05 | <0.00001 | 2.00E-05 |
| **Chr 8** | 0.00032 | 0.00032 | 0.800 | 0.00036 | 4.00E-04 | 2.00E-05 | <0.00001 | 3.00E-05 |
| **Chr 9** | 3.00E-04 | 3.00E-04 | 0.827 | 6.00E-04 | 6.00E-04 | 2.00E-05 | <0.00001 | 3.00E-05 |
| **Chr 10** | 0.00012 | 0.00012 | 0.825 | 4.00E-04 | 0.00042 | 2.00E-05 | <0.00001 | 3.00E-05 |
| **Chr 11** | 0.00036 | 0.00036 | 0.506 | 0.00021 | 0.00027 | 2.00E-05 | <0.00001 | 2.00E-05 |
| **Chr 12** | 0.00042 | 0.00042 | 0.667 | 0.00035 | 0.00033 | 2.00E-05 | <0.00001 | 2.00E-05 |
| **Chr 13** | 0.00032 | 0.00032 | 0.800 | 7.00E-05 | 0.00014 | 2.00E-05 | <0.00001 | 3.00E-05 |
| **Chr 14** | 0.00033 | 0.00033 | 0.801 | 0.00042 | 0.00044 | 0 | <0.00001 | 2.00E-05 |
| **Chr 15** | 0.00036 | 0.00036 | 0.827 | 4.00E-04 | 0.00041 | 2.00E-05 | <0.00001 | 3.00E-05 |
| **Chr 16** | 0.00036 | 0.00036 | 0.825 | 0.00029 | 0.00033 | 2.00E-05 | <0.00001 | 2.00E-05 |
| **Chr 17** | 0.00036 | 0.00036 | 0.825 | 0.00035 | 0.00038 | 2.00E-05 | <0.00001 | 1.00E-05 |
| **Chr 18** | 0.00013 | 0.00013 | 0.838 | 0.00038 | 4.00E-04 | 2.00E-05 | <0.00001 | 2.00E-05 |
| **Chr 19** | 0.00042 | 0.00042 | 0.679 | 4.00E-04 | 4.00E-04 | 2.00E-05 | <0.00001 | 2.00E-05 |
| **Chr 20** | 4.00E-04 | 4.00E-04 | 0.755 | 0.00028 | 0.00033 | 1.00E-05 | <0.00001 | <0.00001 |
| **Chr 21** | 0.00032 | 0.00032 | 0.840 | 4.00E-04 | 4.00E-04 | 2.00E-05 | <0.00001 | 4.00E-05 |
| **Chr 22** | 0.00033 | 0.00033 | 0.493 | 0.00013 | 0.00021 | 2.00E-05 | <0.00001 | 2.00E-05 |
| **Chr X** | 4.00E-04 | 4.00E-04 | 0.722 | 0.00029 | 0.00036 | 2.00E-05 | <0.00001 | 2.00E-05 |
