# Supplementary material for: Telomere lengths in women treated for breast cancer show associations with chemotherapy, pain symptoms, and cognitive domain measures: a longitudinal study
Source: Breast Cancer Res. 2020 Dec 4;22:137. doi: 10.1186/s13058-020-01368-6 (PMC7716505; doi:10.1186/s13058-020-01368-6)
Supplement: Supplementary file 8 — Additional file 8. Chromosome-Specific Telomere Length and Cancer Risk. Literature review of previous reports of chromosome-specific telomere lengths related to cancer. [file 13058_2020_1368_MOESM8_ESM.docx]

**Additional File 8: Chromosome-Specific Telomere Length and Cancer Risk**

| **Reference** | **Study Sample** | **Method** | **Summary of Finding** |
| --- | --- | --- | --- |
| Zheng et al., 2009 [58] | Cases: 153 women with breast cancer who have not been treated with chemotherapy or radiotherapy  Controls: 159 age matched healthy women | Q-FISH | Short telomere on 9p is strongly associate with breast cancer risk |
| Xing et al., 2009 [59] | Cases: 94 untreated patients with esophageal cancer  Controls: 94 age, gender and ethnicity matched healthy subjects | STELA | Short telomere length on 17p and 12q play a more prominent role in the etiology of esophageal cancer than 11q and 2p |
| Zheng et al., 2011 [60] | Cases: 204 women with breast cancer who have not been treated with chemotherapy or radiotherapy  Controls: 236 age matched healthy women | PNA-FISH | Short telomere length on 9p, 15p, 15q and Xp is associated with breast cancer risk in pre-menopausal women |
|  |  |  |  |

Q-FISH= Quantitative Fluorescence In Situ Hybridization

PNA-FISH= Peptide Nucleic Acid Fluorescence In Situ Hybridization

STELA= Single Telomere Length Analysis

**References**

Zheng YL, Loffredo CA, Shields PG, Selim SM. 2009. Chromosome 9 arm-specific telomere length and breast cancer risk. Carcinogenesis 30(8):1380-6.

Xing J, Ajani JA, Chen M, Izzo J, Lin J, Chen Z, Gu J, Wu X. 2009. Constitutive short telomere length of chromosome 17p and 12q but not 11q and 2p is associated with an increased risk for esophageal cancer. Cancer Prev Res (Phila) 2(5):459-65.

Zheng YL, Zhou X, Loffredo CA, Shields PG, Sun B. 2011. Telomere deficiencies on chromosomes 9p, 15p, 15q and Xp: Potential biomarkers for breast cancer risk. Hum Mol Genet 20(2):378-86.
