# Supplementary material for: Telomere lengths in women treated for breast cancer show associations with chemotherapy, pain symptoms, and cognitive domain measures: a longitudinal study
Source: Breast Cancer Res. 2020 Dec 4;22:137. doi: 10.1186/s13058-020-01368-6 (PMC7716505; doi:10.1186/s13058-020-01368-6)
Supplement: Supplementary file 9 — Additional file 9. Studies on the Effect of Doxorubicin (Adriamycin) on Telomere Biology. Literature review of previous reports assessing the biological response/mechanism of Doxorubincin (also called Adriamycin) on telomeres. [file 13058_2020_1368_MOESM9_ESM.docx]

**Additional File 9: Studies on the Effect of Doxorubicin (Adriamycin) on Telomere Biology**

| **Reference** | **Sample Type** | **Study Outcome** |
| --- | --- | --- |
| Elmore et al., 2002 | MCF-7 breast tumor cell line | Breast tumor cells treated with Adriamycin require both functional p53 and telomere dysfunction to exhibit a senescence phenotype |
| Mitchell et al., 2010 | LiSa-2 ALT positive cell line derived from a poorly differentiated pleomorphic liposarcoma  LS2 cell line derived from ALT-positive pleomorphic liposarcoma  SW872 cell line expressing both components of telomerase | Cell lines sensitivity to doxorubicin correlated to topoisomerase 2A (TOP2A) gene expression, with LiSa-2 cell line showing the highest sensitivity to doxorubicin and the highest expression of TOP2A followed by LS2 cell line  SW872 cell line showed the lowest sensitivity to doxorubicin and the lowest expression of TOP2A |
| Buttiglieri et al., 2011 | Mesenchymal stem cells derived from normal human bone marrow | Telomere loss following treatment with doxorubicin that was also associated with defects in proliferation and differentiation |
| Li et al., 2012 | T-lymphocytes isolated from buffy coats of healthy individuals with stimulated telomerase activation  Normal human lung fetal fibroblasts representing telomerase deficient cells | Rapid loss of telomeric DNA sequence, coupled with down-regulation of telomerase activity, repression of hTERT, TPP1 and POT1 (members of the shelterin complex) expression in T-lymphocytes and fibroblasts treated with doxorubicin resulting in telomere dysfunction in a high fraction of cells |
| Kato et al., 2013 | Human cervical carcinoma cell line (HeLa), human normal fibroblast cell line (WI-38), human osteosarcoma cell lines (U-2-OS and Saos-2 | TRF1 and POT1 (members of the shelterin complex) mRNA levels were commonly down-regulated in both cancerous and normal cell lines following treatment with doxorubicin. On the other hand, RAP1 was up-regulated in a time-dependent manner in 2-OS cells only |
| Sanoff et al., 2014 | 33 women with early stage breast cancer (I-III) who were receiving doxorubicin, cyclophosphamide and taxane | Telomere length was measured before chemotherapy and 12 months after chemotherapy, with no significant change observed |

**References**

Elmore LW, Rehder CW, Di X, McChesney PA, Jackson-Cook CK, Gewirtz DA, et al. Adriamycin-induced senescence in breast tumor cells involves functional p53 and telomere dysfunction. J Biol Chem 2002; 277(38):35509-15.

Li P, Hou M, Lou F, Bjorkholm M, Xu D. Telomere dysfunction induced by chemotherapeutic agents and radiation in normal human cells. Int J Biochem Cell Biol **2012**; 44(9):1531-40.

Mitchell MA, Johnson JE, Pascarelli K, Beeharry N, Chiourea M, Gagos S, Lev D, von Mehren M, Kipling D, Broccoli D. 2010. Doxorubicin resistance in a novel in vitro model of human pleomorphic liposarcoma associated with alternative lengthening of telomeres. Mol Cancer Ther 9(3):682-92

Buttiglieri S, Ruella M, Risso A, Spatola T, Silengo L, Avvedimento EV, Tarella C. 2011. The aging effect of chemotherapy on cultured human mesenchymal stem cells. Exp Hematol 39(12):1171-81.

Kato M, Nakayama M, Agata M, Yoshida K. 2013. Gene expression levels of human shelterin complex and shelterin-associated factors regulated by the topoisomerase II inhibitors doxorubicin and etoposide in human cultured cells. Tumour Biol 34(2):723-33.

Sanoff HK, Deal AM, Krishnamurthy J, Torrice C, Dillon P, Sorrentino J, Ibrahim JG, Jolly TA, Williams G, Carey LA, and others. 2014. Effect of cytotoxic chemotherapy on markers of molecular age in patients with breast cancer. J Natl Cancer Inst 106(4):dju057.
