# Supplementary material for: Telomere lengths in women treated for breast cancer show associations with chemotherapy, pain symptoms, and cognitive domain measures: a longitudinal study
Source: Breast Cancer Res. 2020 Dec 4;22:137. doi: 10.1186/s13058-020-01368-6 (PMC7716505; doi:10.1186/s13058-020-01368-6)
Supplement: Supplementary file 10 — Additional file 10. Hypothesized Biological Cascade Contributing to Psychoneurological Symptoms in Women Receiving Chemotherapy for Breast Cancer. Schematic representation of inter-relationships between telomeres and inflammation, which lead to psychoneurological symptoms via alterations in epigenetic patterns/gene expression, and/or senescence. [file 13058_2020_1368_MOESM10_ESM.docx]

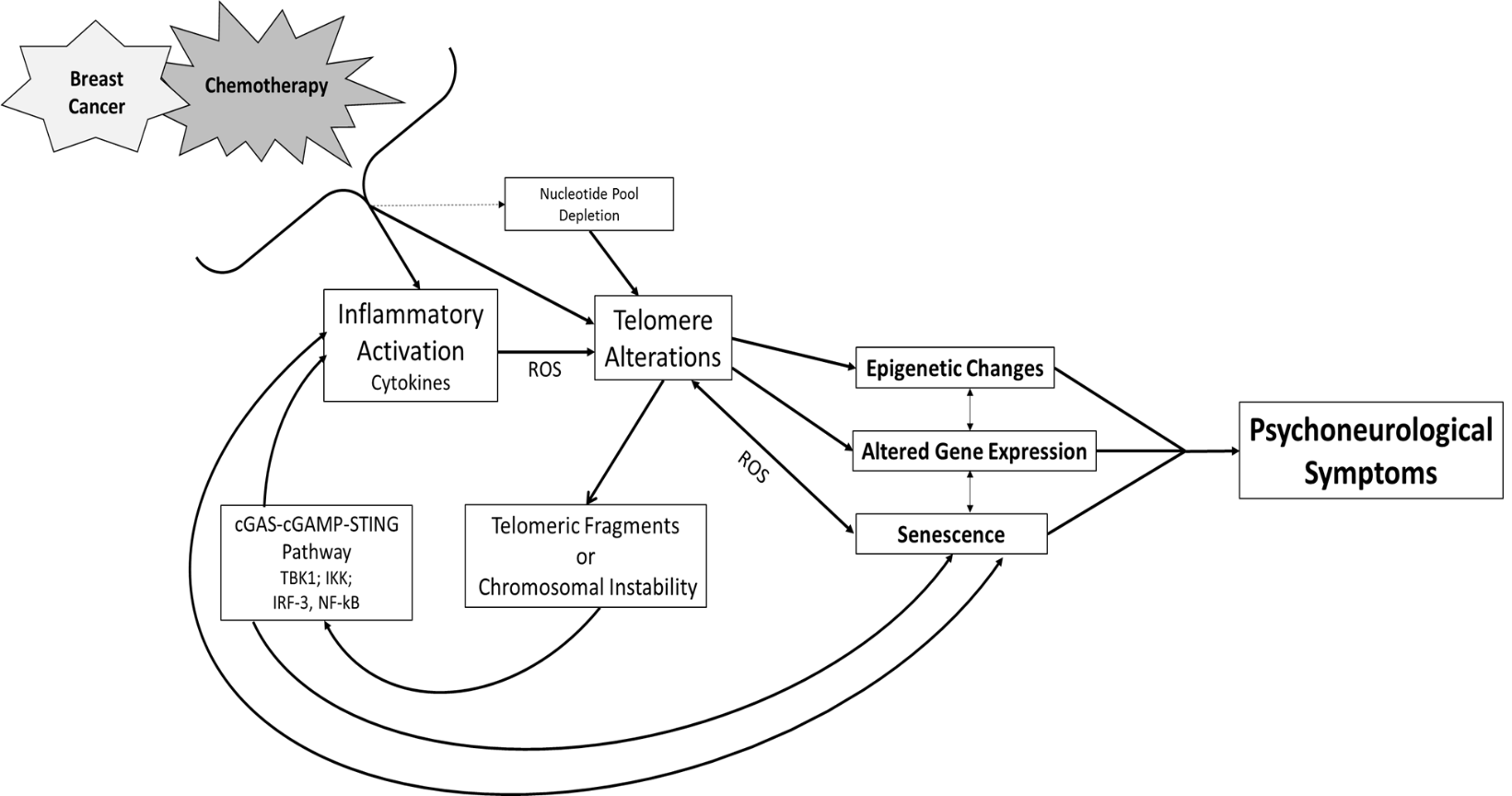


**Additional File 10. Hypothesized Biological Cascade Contributing to Psychoneurological Symptoms in Women Receiving Chemotherapy for Breast Cancer.** Breast cancer treatment (or the cancer itself) can lead to telomere alterations through a variety of influences, with several of these changes also being related to inflammatory activation. Telomere length alterations can also impact subtelomeric gene expression and chromatin compaction, thereby contributing to epigenetic changes. The resulting epigenetic, gene expression, and/or senescence effects could lead to cellular changes that contribute to the development and/or persistence of psychoneurological symptoms (Figure adapted from Lyon, et al [7]).

Abbreviations: cGAS = cyclic guanosine monophosphate (GMP)-adenosine monophosphate (AMP) synthase; STING = Stimulator of Interferon Genes; ROS = reactive oxidative stress; TBK-1 = TANK binding kinase 1; IKK = Inhibitor of Nuclear Factor Kappa-B Kinase; IRF-3 = Interferon Regulatory Factor 3; NF-kB = Nuclear Factor Kappa-B.
